# The *Legionella* Effector SdjA Is a Bifunctional Enzyme That Distinctly Regulates Phosphoribosyl Ubiquitination

**DOI:** 10.1128/mBio.02316-21

**Published:** 2021-09-07

**Authors:** Lei Song, Yongchao Xie, Chuang Li, Lidong Wang, Chunlin He, Yong Zhang, Jingya Yuan, Jingjing Luo, Xi Liu, Yu Xiu, Hang Li, Marina Gritsenko, Ernesto S. Nakayasu, Yue Feng, Zhao-Qing Luo

**Affiliations:** a Department of Respiratory Medicine, Center of Infection and Immunity, Key Laboratory of Organ Regeneration and Transplantation of the Ministry of Education, The First Hospital, Jilin University, Changchun, China; b Beijing Advanced Innovation Center for Soft Matter Science and Engineering, Beijing Key Laboratory of Bioprocess, State Key Laboratory of Chemical Resource Engineering, College of Life Science and Technology, Beijing University of Chemical Technology, Beijing, China; c Department of Biological Sciences, Purdue University, West Lafayette, Indiana, USA; d Biological Science Division, Pacific Northwest National Laboratory, Richland, Washington, USA; University of Illinois at Chicago

**Keywords:** type IV secretion, bacterial virulence, deglutamylase, glutamylation

## Abstract

Legionella pneumophila promotes its survival and replication in phagocytes by actively modulating cellular processes using effectors injected into host cells by its Dot/Icm type IV secretion system. Many of these effectors function to manipulate the ubiquitin network of infected cells, thus contributing to the biogenesis of the *Legionella*-containing vacuole (LCV), which is permissive for bacterial replication. Among these, members of the SidE effector family (SidEs) catalyze ubiquitination of functionally diverse host proteins by a mechanism that is chemically distinct from the canonical three-enzyme cascade. The activity of SidEs is regulated by two mechanisms: reversal of the phosphoribosyl ubiquitination by DupA and DupB and direct inactivation by SidJ, which is a calmodulin-dependent glutamylase. In many L. pneumophila strains, SidJ belongs to a two-member protein family. Its homolog SdjA appears to function differently from SidJ despite the high-level similarity in their primary sequences. Here, we found that SdjA is a bifunctional enzyme that exhibits distinct activities toward members of the SidE family. It inhibits the activity of SdeB and SdeC by glutamylation. Unexpectedly, it also functions as a deglutamylase that reverses SidJ-induced glutamylation on SdeA. Our results reveal that an enzyme can catalyze two completely opposite biochemical reactions, which highlights the distinct regulation of phosphoribosyl ubiquitination by the SidJ effector family.

## INTRODUCTION

Intracellular bacterial pathogens have evolved effective mechanisms to maintain the integrity of host cells, which is essential for their success in colonization. One commonly used mechanism is to repress the expression of key virulence factors once the pathogen has been phagocytosed by host cells. A prominent example is the repression of genes involved in flagellum biogenesis by Salmonella enterica serovar Typhimurium and Legionella pneumophila after entering host cells (1, 2). This regulation is necessary because components of flagella are potent immune ligands detectable by intracellular receptors (3). Regulation of gene expression is often complemented by more subtle and specific mechanisms involved in the use of one virulence factor to regulate the activity of another virulence factor whose activity may damage host cells if left uncontrolled. For example, during its entry into nonphagocytic cells, S. enterica Typhimurium utilizes the guanine nucleotide exchange factor (GEF) SopE to activate Rac1 and CDC42, whose activity is antagonized by the GTPase activation protein (GAP) SptP, leading to restoration of host actin cytoskeleton after successful invasion (4).

To create the *Legionella*-containing vacuole (LCV) permissive for its intracellular replication, L. pneumophila modulates the function of a number of cellular processes by effectors injected into host cells by its Dot/Icm secretion system (5). Not surprisingly, branches of vesicle trafficking are targeted by cohorts of effectors of this intravacuolar pathogen. Rab1, a key regulator of the anterograde transport between the endoplasmic reticulum (ER) and the *cis*-Golgi compartment, is hijacked by SidM/DrrA, LepB, SidD, AnkX, and Lem3, which spatially and temporally regulate its activity by distinct biochemical activities (3, 6–9). Arf1, the regulator for retrograde trafficking between these two organelles is co-opted by the bacterial GEF RalF (10). The control of retromer transport and the endosomal network is imposed by effectors such as RidL and VipD, which target Vps29 and phosphatidylinositol 3-phosphate on endosomes, respectively (11–13). Furthermore, it has been shown that the actin cytoskeleton is modulated by the actin nucleator VipA (14), the protease RavK, which cleaves actin (15), and the phosphatase WipA and kinase LegK2, which target the ARP2/3 complex (16, 17). In some cases, a group of effectors coordinate to regulate a cellular event important for the biogenesis of the LCV. For example, the enrichment of phosphatidylinositol 4-phosphate [PI(4)P] on the LCV is largely attributed to a catalytic cascade composed of MavQ, LepB, and SidF, which function as phosphatidylinositide 3-kinase (18, 19), phosphatidylinositide 4-kinase (20), and phosphatidylinositol-3-phosphatase (21), respectively, to synthesize this lipid from phosphatidylinositol. PI(4)P functions as the anchor for a number of effectors, including the E3 ubiquitin ligases SidC and SdcA (22) and the multifunctional protein SidM (23). In addition, the enrichment of PI(4)P on the LCV makes the lipid composition of its membrane resemble that of the *cis*-Golgi compartment, which may facilitate the interception of vesicles originating from the ER (24). Finally, more than 20 effectors have been shown to hijack the ubiquitin network of host cells by diverse mechanisms. At least 12 effectors function as E3 ubiquitin ligases that cooperate with host E1 and E2 enzymes to modify host and, in some cases, bacterial proteins (25). Several effectors catalyze atypical ubiquitination by mechanisms differing from the canonical three-enzyme cascade (26, 27). Multiple deubiquitinases that hydrolyze an array of different polyubiquitin chains have also been described (28). The use of a large panel of effectors to usurp the host ubiquitin network highlights the importance of ubiquitin signaling in the intracellular life cycle of L. pneumophila.

One unique feature associated with Dot/Icm substrates is the existence of closely similar homologs that form distinct protein families (29). For example, SidC and SdcA share 72% identity and 82% similarity in their primary sequences (29), and both are E3 ubiquitin ligases that modify such proteins as the small GTPase Rab10 (30, 31). Similarly, members of the SidE family catalyze phosphoribosyl ubiquitination of a similar substrate pool by an ADP-ribosylation mechanism induced by a mono-ADP-ribosyltransferase (mART) motif (26, 32). SidE-induced ubiquitination of Rab33b leads to its association with the ER by hijacking Golgi-to-ER retrograde trafficking (33). Given the high-level similarity in their primary sequences, it is not surprising that members of these effector families exhibit almost identical activity. Interestingly, contrary to the conventional view that structurally similar proteins often share common activity, a few recent studies found that highly homologous proteins can perform completely opposite biochemical functions. For example, MavC and MvcA share 62% similarity in their primary sequences, their structures are almost superimposable, and both are capable of deamidating ubiquitin at Gln40 (34). However, these two proteins regulate the activity of the E2 enzyme UBE2N by opposite biochemical activities. Whereas MavC inhibits UBE2N function by catalyzing an atypical monoubiquitination that covalently attaches ubiquitin to Lys82 of UBE2N (27), MvcA antagonizes the inhibition by reversing the modification (35). Similarly, domains conferring phosphodiesterase (PDE) activity in SidE, SdeA, SdeB, and SdeC function to cleave a phosphodiester bond in the reaction intermediate ADP-ribosyl ubiquitin (ADPR-Ub) and attach phosphoribosyl ubiquitin to serine residues of substrate proteins (32, 36). However, structurally similar PDE domains in DupA (also known as SdeD [29]) and DupB function to remove phosphoribosyl ubiquitin from modified proteins (37, 38).

In addition to DupA and DupB, which reverse phosphoribosyl ubiquitination, the activity of the SidEs is regulated by SidJ, a glutamylase that is activated by the eukaryotic protein calmodulin (CaM). Activated SidJ inhibits the ADP-ribosyltransferase activity of SidEs by covalently attaching one or more glutamate moieties on the first glutamate residue of the ExE (where “x” represents any amino acid) element of the mART motif that is essential for ubiquitin activation (39–42). Interestingly, in L. pneumophila strains such as Philadelphia 1, SidJ is one member of a family consisting of two proteins (43). Despite the high-level similarity (57% identity; 74% similarity) between SidJ and SdjA(Lpg2508), the latter cannot suppress the yeast toxicity of SdeA (44). In this study, we set out to determine the function of SdjA by examining its impact on the activity of members of the SidE family. Our results reveal that SdjA is a bifunctional enzyme that exhibits distinctly different activities toward members of the SidE family.

## RESULTS

### SdjA distinctively regulates the activity of the SidE family effectors.

**FIG 1:**
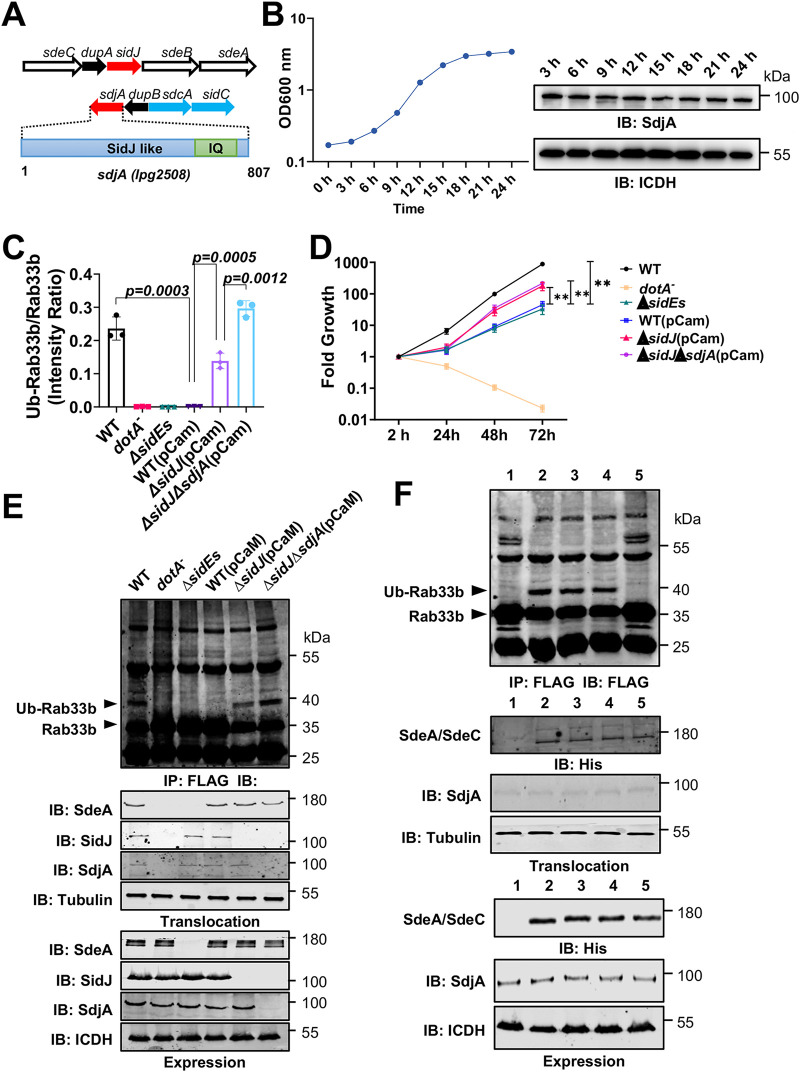
SdjA is constitutively expressed in L. pneumophila and impacts the function of members of the SidE effector family. (A) Gene organization of the loci harboring *sidJ* and *sdjA* on the chromosome of L. pneumophila. Each arrow represents the indicated gene; the enlargement shows the domain architecture of SdjA. The position of the IQ domain for calmodulin binding is indicated. Note that *sidJ* is situated next to *dupA* and *sdjA* is adjacent to *dupB*; both are involved in the regulation of the activity of the SidE family. (B) Expression of *sdjA* in broth-grown bacteria. A saturated culture was diluted 1:20 in fresh medium, and the growth of the culture was monitored by measuring absorbance at 600 nm. Samples with identical cell numbers were withdrawn at the indicated times and probed for SdjA by immunoblotting. The metabolic protein isocitrate dehydrogenase (ICDH) was detected as a loading control. Results are from one of two independent experiments with similar results. (C) Expression of CaM in L. pneumophila affects its virulence. The indicated bacterial strains were used to infect D. discoideum at a multiplicity of infection (MOI) of 0.1, and their intracellular replication was monitored at 24-h intervals for 72 h. Note that expression of CaM affects the virulence of the wild-type strain to a level comparable to that of the mutant lacking the *sidE* effector family. Similar results were obtained in at least three independent experiments, and the results shown are from one representative experiment done in triplicate. **, *P < *0.01. (D and E) Evaluation of the impact of SdjA on the activity of SidEs by expressing CaM in L. pneumophila. Cells transfected to express Flag-Rab33b were infected with the indicated bacterial strains at an MOI of 10 for 2 h. Cell lysates were subjected to immunoprecipitation with agarose beads coated with Flag antibody, and the precipitates were detected by immunoblotting with the Flag antibody. Ubiquitinated Flag-Rab33b from three independent experiments was quantitated with ImageJ (D). Images from a representative experiment are shown (E). Note that expression of CaM in the Δ*sidJ* mutant led to detectable Rab33b ubiquitination, which suggests that the CaM-mediated activity of endogenous SdjA does not completely block the activity of SidEs. The expression and translocation of SdeA, SidJ, and SdjA were probed with the appropriate antibodies, with ICDH and metabolic glyceraldehyde 3-phosphate dehydrogenase (GAPDH) being probed as loading controls. (F) SdjA differently impacts the activity of SdeA and SdeC. Ubiquitination of Flag-Rab33b was evaluated as described for panel E in cells infected with the indicated L. pneumophila strains. Modified Flag-Rab33b was judged by a shift in its molecular weight (top). The expression (lower three panels) and translocation (middle three panels) of SdeA, SdeC, and SdjA were probed by immunoblotting with the appropriate antibodies. ICDH and GAPDH were detected as loading controls. Bacterial strains: 1, Δ*sidE* Δ*sidJ*(vector, vector); 2, Δ*sidE ΔsidJ*(pSdeA, vector); 3, Δ*sidE ΔsidJ*(pSdeA, pCaM); 4, Δ*sidE ΔsidJ*(pSdeC, vector); 5, Δ*sidE ΔsidJ*(pSdeC; pCaM).

Members of the SidE effector family (SidE, SdeA, SdeB, and SdeC) utilize identical biochemical mechanisms to catalyze phosphoribosyl ubiquitination of a large pool of substrates, ranging from Rab small GTPases to reticulons that are involved in ER structure (26, 32). The activity of these ubiquitin ligases is inhibited by SidJ, a calmodulin-dependent glutamylase that catalyzes polyglutamylation on one of the catalytic glutamate residues of their mART motif (39–42). On the chromosome of the Philadelphia 1 strain, while *sidJ* is localized between *sdeB* and *dupA*, which codes for one of the deubiquitinases that function to remove phosphoribosyl ubiquitin from modified substrates, *sdjA* is situated next to *dupB*, which is functionally equivalent to *dupA* (37, 38) (Fig. 1A). Paradoxically, despite sharing 74% similarity in their primary sequences (Fig. S1), SidJ and SdjA seem to differ in their activity, as SdjA is unable to suppress the yeast toxicity of SdeA (44).

10.1128/mBio.02316-21.1 FIG S1 Sequence alignment of SidJ and SdjA. The alignment was performed with Jalview. Identical residues are highlighted with a dark purple background. Download FIG S1, PDF file, 0.3 MB. Copyright © 2021 Song et al. 2021 Song et al. https://creativecommons.org/licenses/by/4.0/ This content is distributed under the terms of the Creative Commons Attribution 4.0 International license.

To determine the function of SdjA, we first examined whether this protein is made by L. pneumophila. Using polyclonal antibodies specific for SdjA, we probed its expression in bacteria grown at different phases in buffered yeast extract medium and found that SdjA is expressed at similar levels throughout the growth cycle (Fig. 1B). This expression pattern is similar to that of its homolog SidJ (43), suggesting that both SdjA and SidJ are made and translocated into host cells likely during the entire intracellular life cycle of L. pneumophila.

The requirement of CaM for the glutamylase activity of SidJ predicts that expression of this cofactor in L. pneumophila inactivates endogenous SidEs and phenocopies the Δ*sidE* mutant in intracellular replication (26, 45). Indeed, wild-type L. pneumophila harboring a plasmid expressing CaM displayed a significant growth defect in Dictyostelium discoideum (Fig. 1C). This defect was less severe than that in the Δ*sidE* mutant (lacking all members of the SidE family) (Fig. 1C), suggesting that a portion of proteins of the SidE family remain active. Furthermore, whereas robust ubiquitination of Rab33b occurred in cells infected with the wild-type strain, such modification became undetectable in samples infected with the strain expressing CaM (Fig. 1D and E).

Unlike its ortholog *sidJ*, deletion of *sdjA* does not affect intracellular bacterial replication (43); we therefore examined its impact on the activity of SidEs by expressing CaM in the Δ*sidJ* mutant. As expected, intracellular growth of the Δ*sidJ* Δ*sdjA*(pCaM) strain was similar to that of the Δ*sidJ*(pCaM) strain; both were slightly defective (Fig. 1C). Because *sidJ* is required for maximal intracellular growth (43), it is difficult to distinguish the exact factors directly responsible for the defects observed in the Δ*sidJ*(pCaM) strain. We thus examined SidEs-induced ubiquitination of Rab33b by these L. pneumophila strains. Compared to samples infected with the wild-type strain expressing CaM, the amount of ubiquitinated Rab33b markedly increased in cells infected with the Δ*sidJ*(pCaM) strain and further increased in cells with the Δ*sidJ* Δ*sdjA*(pCaM) strain (Fig. 1D and E). These results suggest that endogenous SdjA inhibits the activity of a fraction of SidE family proteins or only some of its members.

We further examined the impact of SdjA on members of the SidE family by coexpressing CaM with SdeA or SdeC in the Δ*sidE* Δ*sidJ* strain. As expected, introduction of a SdeA-expressing plasmid into this mutant restored its ability to ubiquitinate Rab33b and coexpression of CaM did not affect such modification (Fig. 1F, strains 2 and 3). This result is consistent with the notion that SdjA cannot inactivate SdeA even in the presence of CaM. In contrast, whereas introduction of SdeC can similarly restore the ability of the Δ*sidE* Δ*sidJ* strain to ubiquitinate Rab33b, coexpression of CaM led to abolishment of such modification (Fig. 1F, strains 4 and 5). The expression and translocation of SdeA, SdeC, or SdjA was similar among the relevant L. pneumophila strains (Fig. 1F, bottom), indicating that the change in the function of SdeC likely is caused by the activity of SdjA.

### SdjA selectively suppresses the yeast toxicity of members of the SidE family.

**FIG 2:**
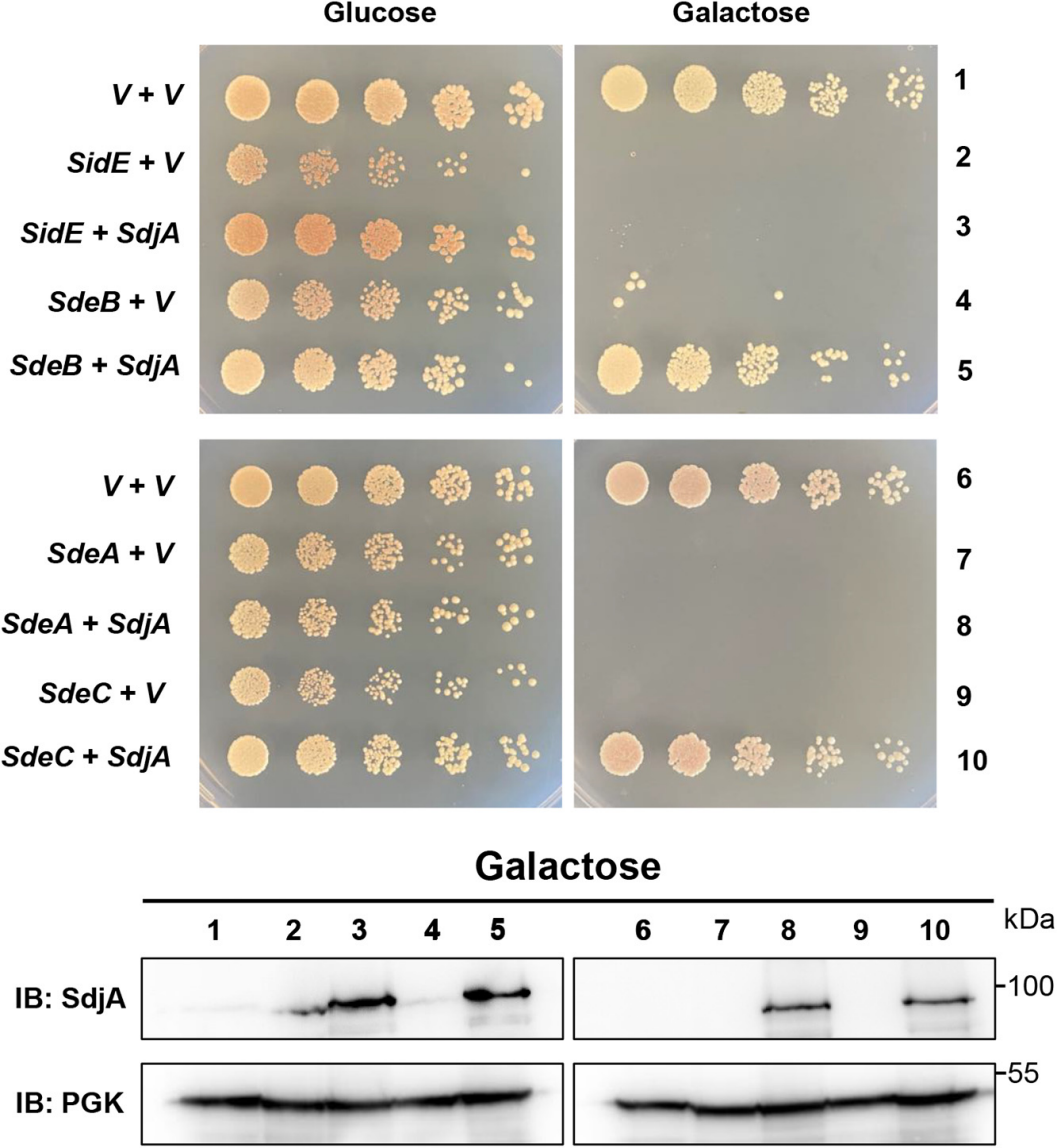
SdjA selectively rescues the yeast toxicity of members of the SidE family. A plasmid that expresses SdjA from the glyceraldehyde 3-phosphate dehydrogenase promoter was introduced into yeast strains harboring SidE, SdeA, SdeB, or SdeC expressed from a galactose-inducible promoter. Similar amounts of yeast cells were diluted and spotted onto medium supplemented with glucose or galactose. Images of the plates were acquired 4 days after incubation at 30°C (top). The expression of *sdjA* in these yeast strains was detected after galactose induction. The metabolic enzyme 3-phosphoglycerate kinase (PGK) was probed as a loading control (bottom).

Unlike SidJ, which effectively suppresses yeast toxicity of each member of the SidE family (46), SdjA was unable to alleviate the toxicity of SdeA (44). The phenotypes associated with L. pneumophila strains expressing CaM suggest that SdjA differently impacts the activity of members of the SidE family (Fig. 1F); we therefore further analyzed such differences by determining its ability to counteract the yeast toxicity of each SidE family member. Consistent with the results from L. pneumophila strains expressing CaM, SdjA effectively suppressed the yeast toxicity caused by SdeB or SdeC. In contrast, although expressed at similar levels, SdjA cannot rescue the growth of strains expressing SidE or SdeA (Fig. 2). These results further support the notion that SdjA differently impacts the activity of members of the SidE family.

### SdjA inhibits the ubiquitin ligase activity of SdeB and SdeC but not SidE and SdeA.

**FIG 3:**
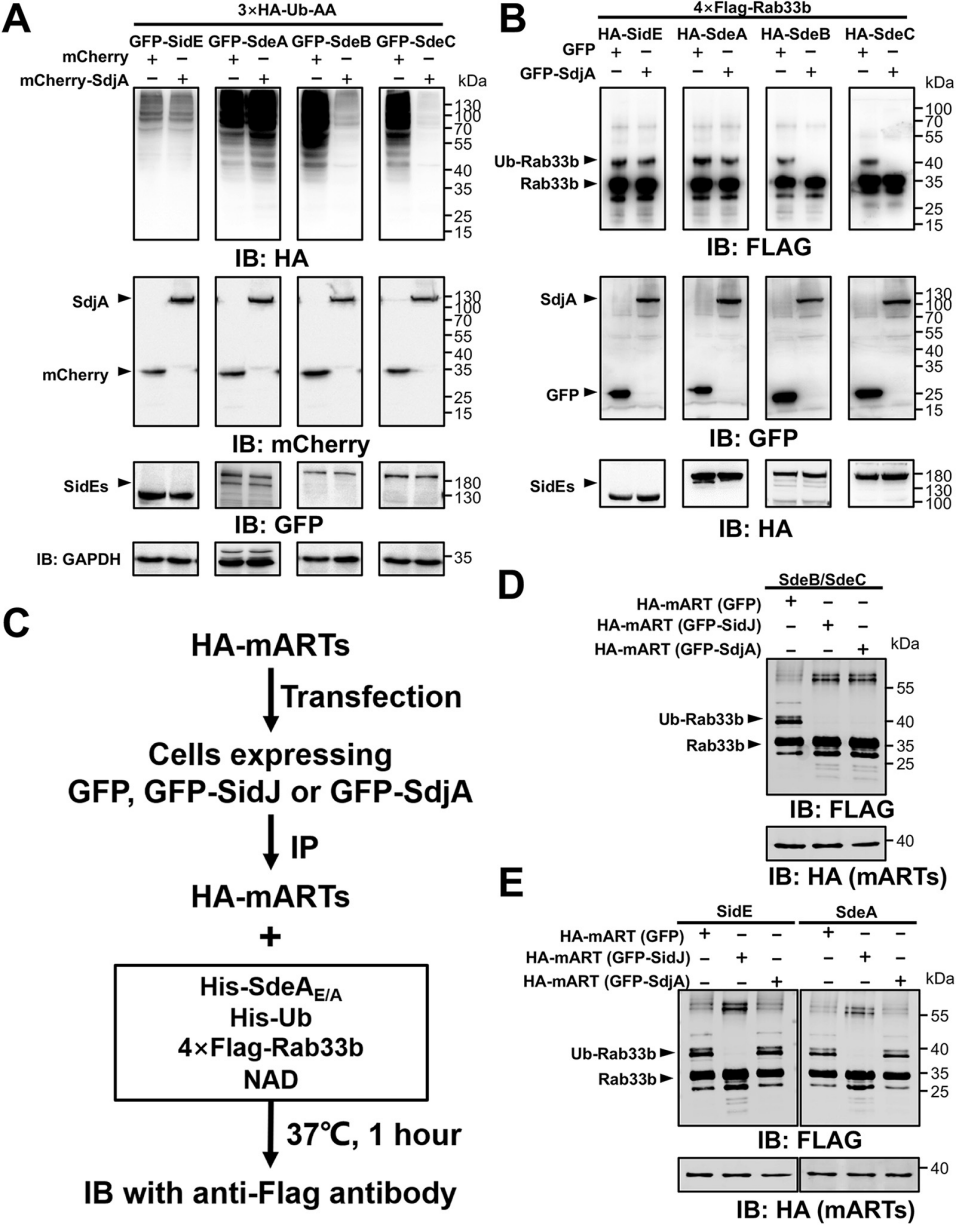
SdjA interferes with the ubiquitin ligase activity of SdeB and SdeC but not SidE or SdeA. (A) HEK293 cells were transfected to express GFP-tagged SidE family effectors together with mCherry-tagged SdjA and 3×HA-Ub-AA for 18 h. Total proteins resolved by SDS-PAGE were probed with the HA antibody to evaluate ubiquitination induced by SidEs. The expression of SdjA (by mCherry-specific antibodies) and the testing of SidE family effectors (by GFP-specific antibodies) were examined. GAPDH was probed as a loading control. Note that SdjA did not interfere with the expression of SdeB or SdeC but effectively inhibited ubiquitination induced by these proteins. (B) Rab33b ubiquitination induced by SdeB and SdeC is inhibited by SdjA. HEK293 cells were transfected with plasmids that direct the expression of Flag-Rab33b, SdjA, and HA-tagged members of the SidE family. Ubiquitination of Rab33b was indicated by the increase of its molecular weight in immunoblotting with the Flag antibody. The expression of relevant proteins was detected with appropriate antibodies. Data shown are from one representative of three independent experiments with similar results. (C) Diagram of the experimental procedure used to determine the impact of SidJ or SdjA on the activity of the mART domains from members of the SidE family. HA-tagged mART domains from members of the SidE family were coexpressed with GFP, GFP-SidJ, or GFP-SdjA in mammalian cells by transfection. The mART domains isolated by immunoprecipitation with agarose beads coated with HA-specific antibody were incubated with NAD and ubiquitin to produce ADPR-Ub, followed by modification of Rab33b with the SdeA_E/A_ mutant. SidJ with the ability to inhibit the activity of all members of the SidE family was used as a control. (D) The mART domain of SdeB (or SdeC) coexpressed with SdjA lost the activity to produce ADPR-Ub and thus was unable to ubiquitinate Rab33b with SdeA_E/A_. (E) Coexpression of the mART domain of SdeA or SidE with SdjA did not affect their ability to produce ADPR-Ub. Note the production of Ub-Rab33b with SdeA_E/A_. In contrast, SidJ inactivates such activity.

Members of the SidE family induce ubiquitination in which ubiquitin is covalently linked to a protein substrate via Arg42 through a phosphoribosyl moiety (32, 36). To determine how SdjA impacts the function of these ubiquitin ligases, we coexpressed in mammalian cells each of them with SdjA and HA-Ub-AA, a ubiquitin mutant in which the two carboxyl-terminal glycine residues have been replaced with alanine. HA-Ub-AA can be used only by SidEs, not by enzymes of the canonical ubiquitination machinery, thus reducing background ubiquitination when probed with the HA antibody. With the exception of SidE, which exhibited considerably lower activity, expression of Ub-AA with SdeA, SdeB, or SdeC led to robust ubiquitination by HA-Ub-AA (Fig. 3A). When SdjA was coexpressed, modification by Ub-AA induced by SidE or SdeA was not detectably decreased. In contrast, in experiments with SdeB or SdeC, coexpression of SdjA drastically reduced the amounts of modified proteins (Fig. 3A). We further examined the differential impact of SdjA on these ubiquitin ligases by testing the modification of Rab33b. Consistent with the results from probing ubiquitination in total proteins, SdjA interferes with Rab33b ubiquitination induced by SdeB or SdeC but not by SidE or SdeA (Fig. 3B).

SidJ exerts its inhibitory effect on the activity of SidEs by catalyzing covalent attachment of one or more glutamate residues onto a glutamate critical for NAD binding in the mART motif (39–42). We thus examined whether the mART domain in these ubiquitin ligases is sufficient to allow the selective inhibition imposed by SdjA. Because the mART domains from SdeB and SdeC are virtually identical (differing by only one residue) (Fig. S2), we used the one from SdeB(541–886) along with those from SidE(554–898) and SdeA(556–903) in our experiments. Each of these three mART domains was coexpressed with SdjA or SidJ in mammalian cells, and the corresponding mART-containing proteins isolated by immunoprecipitation were incubated with a reaction cocktail that allows Rab33b ubiquitination (Fig. 3C). In each case, the mART domain that never encountered SidJ or SdjA robustly induced Rab33b ubiquitination. In contrast, all mART domains coexpressed with SidJ lost the activity (Fig. 3D and E). While the mART domain of SdeB coexpressed with SdjA lost the ability to induce Rab33b ubiquitination, similarly prepared mART domains from SidE and SdeA maintained robust activity (Fig. 3D and E). Thus, the mART domains of SdeA and SdeB have sufficient variations to confer the distinct responses to the activity of SdjA.

10.1128/mBio.02316-21.2 FIG S2 Sequence alignment of the mART domains from SdeB and SdeC. The alignment was generated with Jalview, identical residues are shown in a dark purple background and the glutamate residues attacked by SdjA are white letters in a red background. Download FIG S2, PDF file, 0.09 MB. Copyright © 2021 Song et al. 2021 Song et al. https://creativecommons.org/licenses/by/4.0/ This content is distributed under the terms of the Creative Commons Attribution 4.0 International license.

### SdjA is a CaM-dependent glutamylase against SdeB and SdeC.

**FIG 4:**
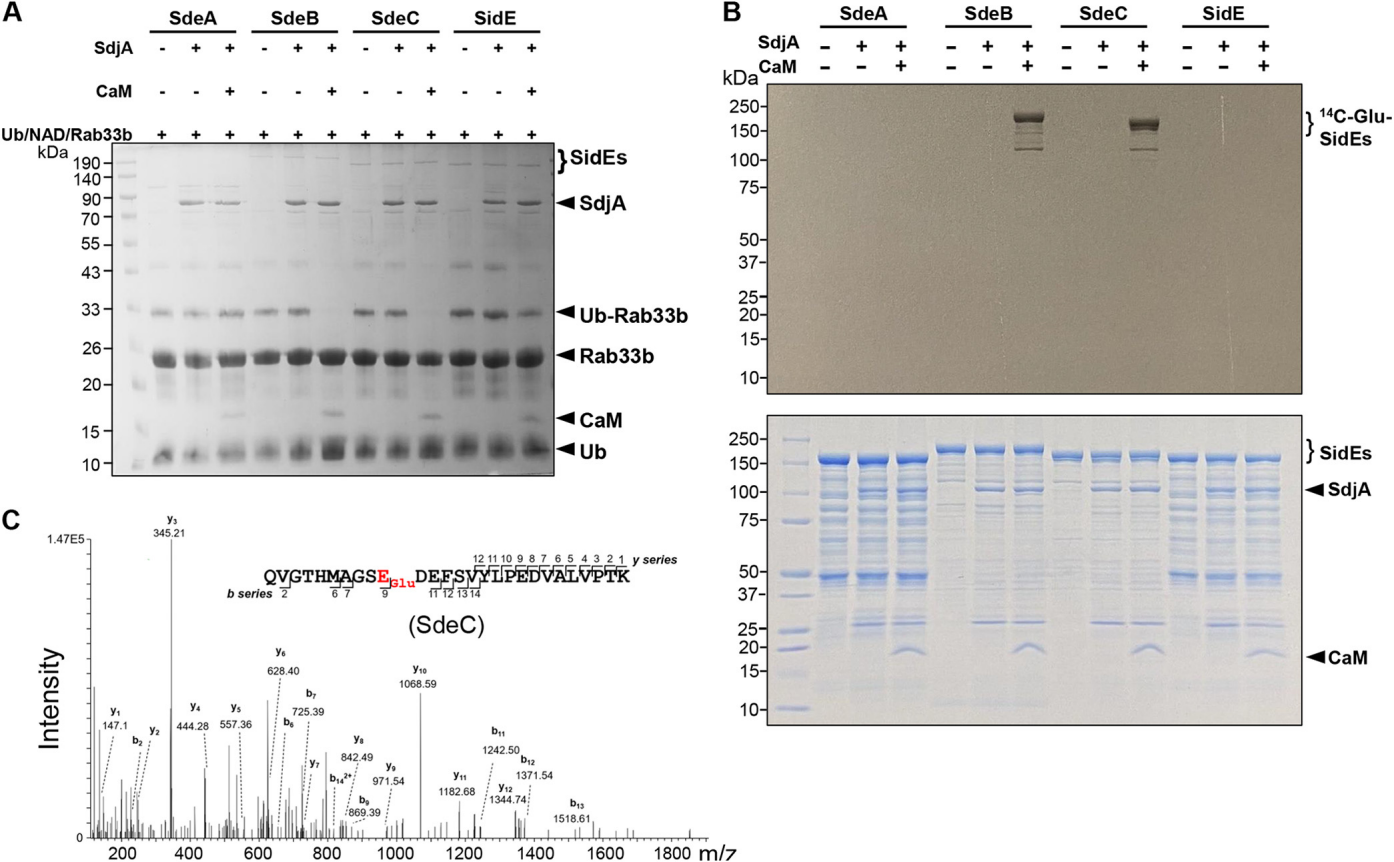
SdjA attacks the mART motif of SdeB and SdeC by glutamylation. (A) Inhibition of SdeB and SdeC by SdjA requires CaM. Recombinant SdjA was incubated with glutamate and each protein of the SidE family with or without CaM for 2 h at 37°C. The activity of these ubiquitin ligases was assessed by adding a reaction mixture containing ubiquitin, NAD, and Rab33b. Note the loss of activity by SdeB and SdeC in the presence of CaM (middle two sets of samples). (B) Transfer of glutamate to SdeB and SdeC by SdjA. Recombinant SdjA was incubated with each protein of the SidE family in reaction mixtures containing [^14^C]glutamate with or without CaM at 37°C for 2 h. After SDS-PAGE, gels stained with Coomassie brilliant blue (bottom) were dried, and the incorporation of radiolabeled glutamate into these ubiquitin ligases was detected by autoradiograph (top). Note the CaM-dependent modification of SdeB and SdeC. (C) SdjA induced glutamylation on Glu843 within the mART motif of SdeC. His_6_-SdeC was incubated with SdjA, glutamate, and CaM. The protein band corresponding to His_6_-SdeC was excised from SDS-PAGE gels and analyzed by mass spectrometry, which detected a glutamylation in the fragment Q_834_VGTHMAGSEDEFSVYLPEDVALVPTK_860_. The tandem mass (MS/MS) spectrum shows the fragmentation profile of the modified peptide Q_834_VGTHMAGSE_Glu_DEFSVYLPEDVALVPTK_860_, including ions b_9_ and b_11_, which confirms the modification site at the Glu843 residue.

The inhibitory effect of SdjA on SdeB and SdeC resembles that of SidJ; we therefore examined whether the activity of SdjA against the mART activity of SdeB (SdeC) requires CaM. Reactions involving members of the SidE family, SdjA, and glutamate with or without CaM were allowed to proceed for 2 h at 37°C, and then the activity of SidE family proteins was tested by adding a cocktail that allows them to ubiquitinate Rab33b. In reactions that involved SdeA, SdjA together with CaM did not detectably affect its ubiquitin ligase activity. In contrast, the activity of SdeB or SdeC was inhibited by SdjA in a CaM-dependent manner, which is consistent with results from a recent report (47). The inhibitory effect against SidE was detectable but was not as robust as that seen with SdeB or SdeC (Fig. 4A). Thus, similar to SidJ, the glutamylase activity of SdjA against SdeB and SdeC requires the cofactor CaM.

Consistent with the results from measuring its inhibitory effects on the ubiquitin ligase activity, in reactions using [^14^C]glutamate, we detected [^14^C]Glu-labeled SdeB or SdeC by SdjA in a CaM-dependent manner. Under similar reaction conditions, [^14^C]Glu-labeled SdeA or SidE was not detectable by autoradiograph despite extended exposure time (Fig. 4B).

Mass-spectrometric analysis revealed that SdjA catalyzes the attachment of a glutamate moiety to the first glutamate residue of the ExE element in the mART motifs of SdeB and SdeC. Although SdeB and SdeC differ greatly in length (29, 48), the modified residue in each case is Glu843 (Fig. 4C and Fig. S3), which is also the site modified by SidJ (39–42). Together, these results establish that SdjA inhibits the activity of SdeB and SdeC by glutamylation in a manner similar to that of SidJ.

10.1128/mBio.02316-21.3 FIG S3 Identification of the SdjA-induced glutamylation site on SdeB. Modified His_6_-SdeB in reaction mixtures containing SdjA, glutamate and CaM was analyzed by mass spectrometry which detected a glutamylation in the fragment Q_834_VGTHMTGSEDEFSVYLPEDVALVPTK_860_. Tandem mass (MS/MS) spectrum shows the fragmentation profile of the modified peptide –Q_834_VGTHM_ox_TGSE_Glu_DEFSVYLPEDVALVPTK_860_, including ions b_9_ and y_14_, which shows that the modification site is at either the Glu843 or Glu845 residue. Oxidation on Met839 was also detected. Download FIG S3, PDF file, 0.03 MB. Copyright © 2021 Song et al. 2021 Song et al. https://creativecommons.org/licenses/by/4.0/ This content is distributed under the terms of the Creative Commons Attribution 4.0 International license.

### SdjA is a deglutamylase that reverses the SidJ-induced glutamylation of SdeA.

**FIG 5:**
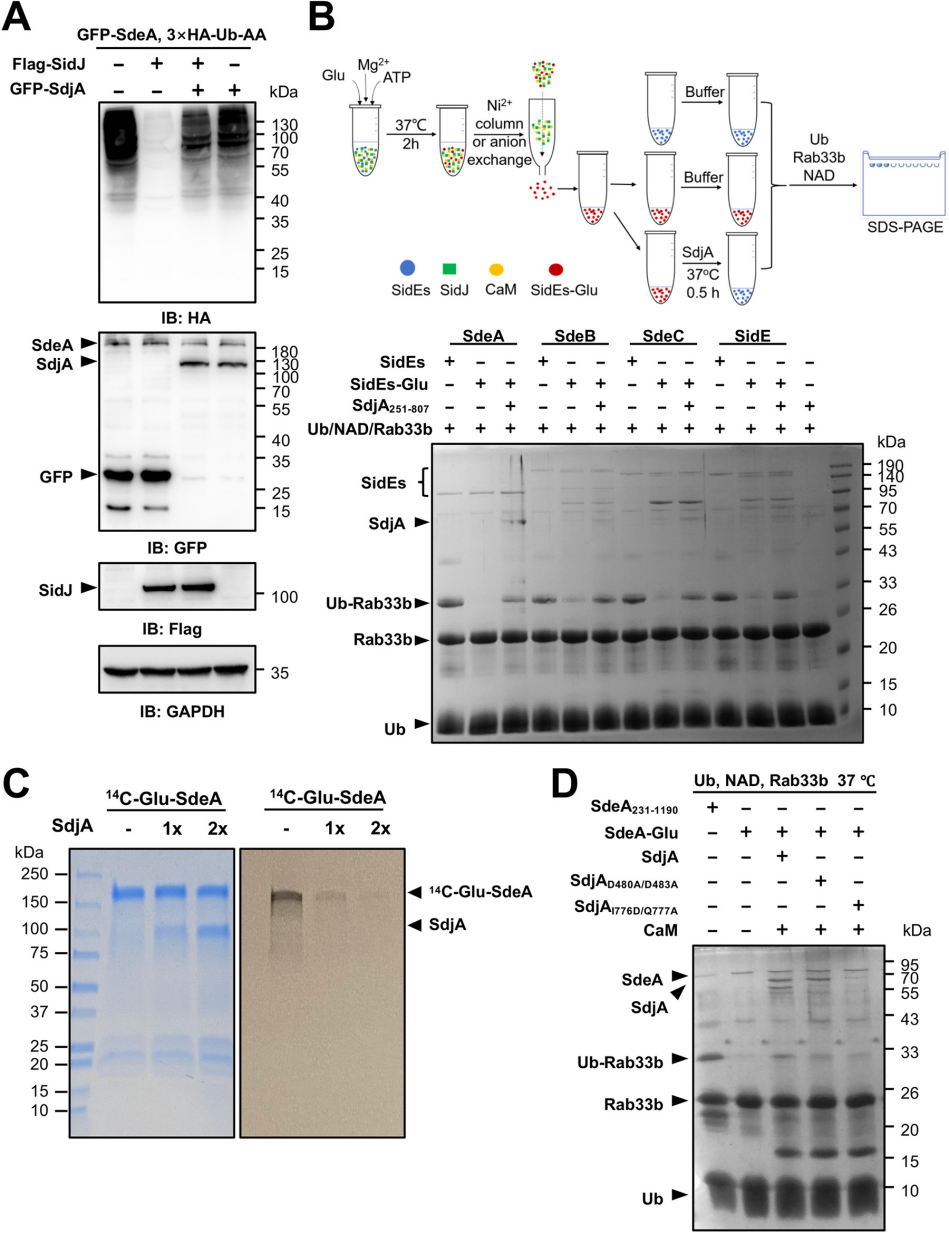
SdjA functions as a deglutamylase to reverse the modification induced by SidJ on SdeA. (A) SdjA antagonizes the inhibitory effects of SidJ on the ubiquitination activity of SdeA. HEK293 cells were transfected with the indicated plasmid combinations, and ubiquitination by HA-Ub-AA was probed by immunoblotting with the HA-specific antibody (top). The expression of relevant proteins was probed with the appropriate antibodies (middle and bottom). Note that coexpression of SdjA with SidJ restored ubiquitination induced by SdeA (compared the second and third lanes). (B) A central fragment of SdjA (SdjA_251–807_) exhibited deglutamylase activity. Proteins of the SidE family were individually glutamylated by SidJ following a procedure depicted in the top panel. In each case, glutamylated protein was produced by reactions that contain SidJ, ATP, and glutamate. Modified proteins were purified; one half of each modified protein was incubated with SdjA_251–807_ prior to being assayed for the ubiquitin ligase activity with a cocktail that contained NAD, ubiquitin, and Rab33b, and the second half was used directly in the activity assay. For each member, a reaction with native active protein was included as a control. Note that SdjA_251–807_ restored the activity of all members of the SidE family that had been inactivated by SidJ as indicated by the formation of Ub-Rab33b (compare the second and third lanes for each protein) (bottom). (C) Deglutamylation of [^14^C]Glu-SdeA by GST-SdjA_37–782._ [^14^C]Glu-SdeA isolated from a reaction mixture containing SidJ, CaM, SdeA, and [^14^C]Glu was incubated with two different amounts of GST-tagged SdjA for 2 h at 37°C. Samples resolved by SDS-PAGE were subjected to autoradiography (top) and the proteins were detected by Coomassie brilliant blue staining (bottom). (D) A SdjA mutant defective in glutamylase activity retains deglutamylase activity. Glutamylated SdeA obtained by a procedure described in panel B was incubated with SdjA or its mutant defective in glutamylase activity or in the IQ motif prior to being assayed for the ubiquitin ligase activity with reagents listed at the top. Note that SdjA_D480A/D483A_, defective in the pseudokinase domain essential for the glutamylase activity, retains the ability to restore the activity of Glu-SdeA (fourth lane).

The inability to detect the inhibitory effect of SdjA against SdeA prompted us to hypothesize that it may synergize with SidJ. Consistent with results from earlier experiments, SidJ but not SdjA effectively blocked the activity of SdeA (Fig. 5A, second lane). Unexpectedly, in samples expressing both SidJ and SdjA, we observed robust SdeA-induced ubiquitination in multiple independent experiments despite normal SidJ expression (Fig. 5A, third lane).

In light of our earlier finding that the two highly similar proteins, MavC and MvcA, possess completely opposite biochemical activity to regulate the activity of the E2 ubiquitin conjugation enzyme UBE2N (27, 35), we considered the possibility that SdjA inhibits the activity of SidJ, probably by reversing the modification on SdeA. To test this hypothesis, we purified glutamylated SdeA (Glu-SdeA) and used the restoration of its ubiquitin ligase activity to measure the effect of SdjA. Because recombinant SdjA expressed in Escherichia coli tended to be degraded and was not homogenous, we purified full-length SdjA coexpressed with CaM, which allowed us to obtain homogenous SdjA in complex with the cofactor. Unexpectedly, incubation of the SdjA-CaM complex with Glu-SdeA did not restore its ubiquitin ligase activity (Fig. S4, third lane). However, in reactions using a degradation product of SdjA with a molecular weight of approximately 70 kDa obtained from an E. coli strain that did not express CaM, SdeA activity became detectable (Fig. S4, fourth lane), suggesting potential deglutamylation by this fragment of SdjA.

10.1128/mBio.02316-21.4 FIG S4 Full-length SdjA in complex with CaM did not have detectable deglutamylase activity. The SdjA-CaM complex purified from an E. coli strain coexpressing these two proteins or His_6_-SdjA obtained from an E. coli strain without CaM was incubated with enzymatically inactive Glu-SdeA for 30 min at 37°C. The activity of SdeA was assayed by adding a mixture containing NAD, ubiquitin and Rab33b. The formation of Ub-Rab33b was detected by an increase in its molecular weight after Coomassie brilliant blue staining. Native SdeA was included as a control. Similar results were obtained in at least three independent experiments. Download FIG S4, PDF file, 0.2 MB. Copyright © 2021 Song et al. 2021 Song et al. https://creativecommons.org/licenses/by/4.0/ This content is distributed under the terms of the Creative Commons Attribution 4.0 International license.

Next, we constructed several truncation mutants of SdjA and tested their deglutamylase activity. A fragment of SdjA that spans residues 251 to 807 (SdjA_251–807_) expressed in an E. coli strain without CaM was found to effectively restore the ligase activity of Glu-SdeA (Fig. 5B, bottom, third lane). Furthermore, incubation of SdjA_251–807_ with inactive glutamylated SdeB, SdeC, or SidE also led to restoration of their activity (Fig. 5B). In contrast, neither SidJ nor its N-terminal truncated mutant exhibited detectable deglutamylase activity when incubated with these proteins (Fig. S5), suggesting that the deglutamylase activity is specific to SdjA.

10.1128/mBio.02316-21.5 FIG S5 SidJ does not have detectable deglutamylase activity. SidJ (A) or its NTD-truncated form (B) was added to reaction mixtures containing glutamylated SidE, SdeA, SdeB or SdeC. After incubation at 37°C for 30 min, a cocktail containing NAD, ubiquitin and Rab33b was added into each reaction. The formation of Ub-Rab33b was detected as described for Fig. S4. In each case, reaction mixtures that contained native proteins were used as positive controls. Download FIG S5, PDF file, 0.9 MB. Copyright © 2021 Song et al. 2021 Song et al. https://creativecommons.org/licenses/by/4.0/ This content is distributed under the terms of the Creative Commons Attribution 4.0 International license.

To further validate the deglutamylase activity of SdjA, we prepared glutamylated SdeA with [^14^C]Glu and used it in a subsequent assay. Incubation of [^14^C]Glu-SdeA with glutathione *S*-transferase (GST)-tagged SdjA_37–782_ (also containing degradation forms of SdjA) led to removal of [^14^C]Glu from modified SdeA in a dose-dependent manner. Consistent with results from the activity-based assay, CaM is not required for the deglutamylase activity (Fig. 5C).

Similar to SidJ, glutamylation by SdjA requires the pseudokinase domain involved in ATP hydrolysis to activate the target glutamate by acyl adenylation (47). We attempted to separate the two activities of SdjA by replacing Asp_480_ and Asp_483_ with alanine, a manipulation known to abolish its glutamylase activity (47). This mutant retained the ability to deglutamylate Glu-SdeA (Fig. 5D). Furthermore, in line with the observation that CaM is not required for the deglutamylase activity, mutations that eliminate the IQ motif responsible for binding CaM did not impact its ability to remove glutamate from Glu-SdeA (Fig. 5D). Thus, the glutamylase and deglutamylase activities of SdjA are independent, and each is likely catalyzed by a unique catalytic center.

### The deglutamylase activity of SdjA impacts the intracellular life cycle of L. pneumophila.

**FIG 6:**
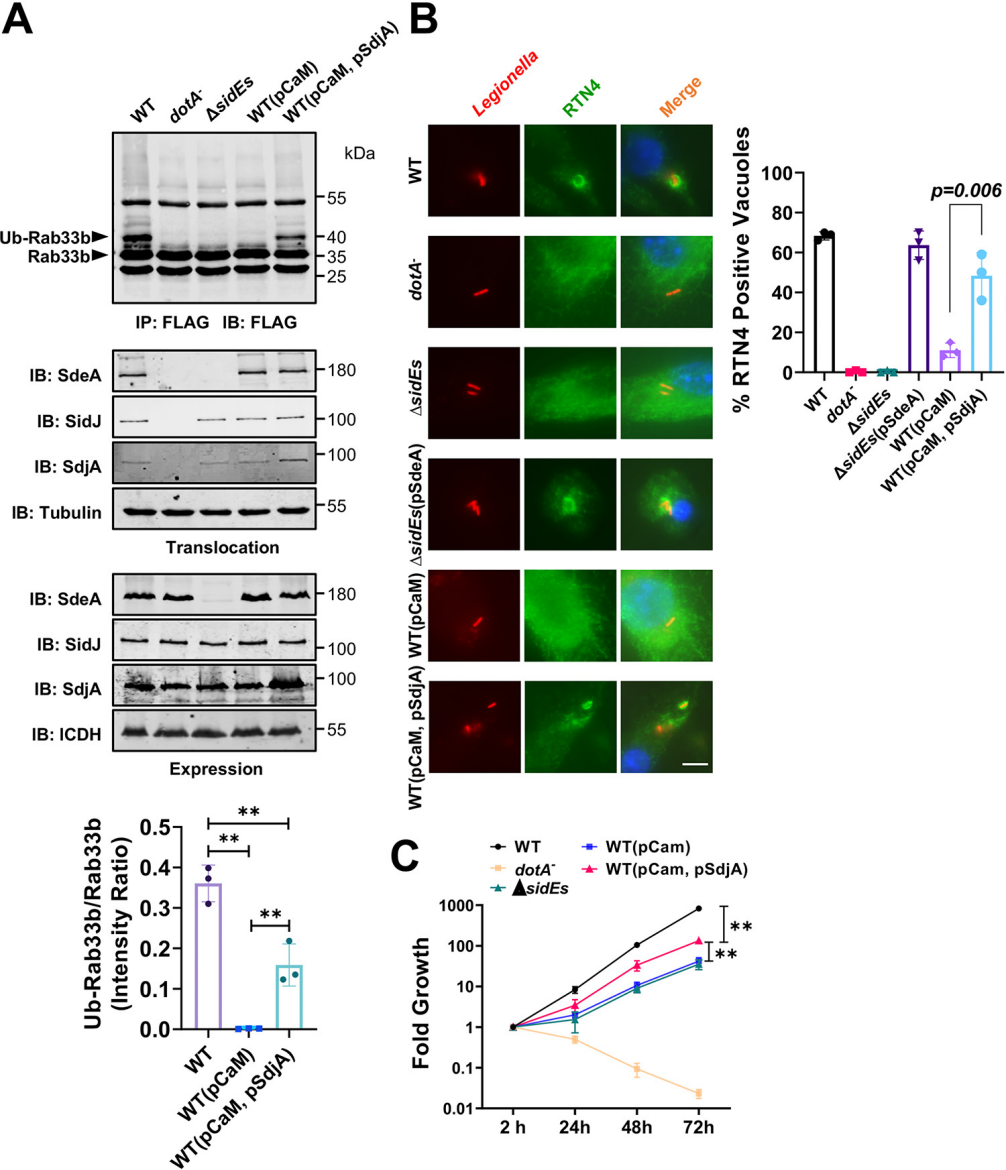
The deglutamylase activity of SdjA impacts the activity of SidEs in cells infected with L. pneumophila. (A) SdjA alleviates CaM-induced inactivation of SidE family effectors. The indicated L. pneumophila strains were used to infect cells transfected to express Flag-Rab33b for 2 h. Lysates of infected cells were subjected to immunoprecipitation with agarose beads coated with Flag antibody, and the precipitates resolved by SDS-PAGE were probed by immunoblotting with the Flag antibody. Ubiquitinated Rab33b was distinguished by an increase in molecular weight. Note the restoration of Rab33b ubiquitination by SdjA in the wild-type strain expressing CaM (fifth lane). The expression (lower four panels) and translocation (middle four panels) of the relevant proteins was probed with ICDH and tubulin as loading control, respectively. Quantitation of the modified Rab33b was obtained by measuring the intensity of the modified bands in three independent experiments against the nonspecific band lower than Flag-Rab33b (top right). Data are means and standard errors (SE). **, *P < *0.01. (B) Impact of SdjA evaluated by the recruitment of RTN4 by the bacterial phagosome. The indicated bacterial strains were used to infect bone marrow-derived macrophages (BMDMs) at an MOI of 10 for 6 h. Fixed infected cells were sequentially stained with antibodies specific for L. pneumophila and RTN4. The recruitment of RTN4 to the LCV was inspected and counted under an Olympus IX-81 microscope. At least 300 phagosomes were counted for each sample. Results shown are from three independent experiments done in triplicate and are means and SE. (C) SdjA impacts intracellular replication of L. pneumophila. The indicated bacterial strains were used to infect D. discoideum at an MOI of 1, and the growth of the bacteria was monitored by plating lysates of infected cells on bacteriological medium at the indicated time points. Note that introduction of SdjA into strain Lp02(pCaM) promotes its growth in this host. **, *P < *0.01.

Next, we examined the effects of the deglutamylase activity of SdjA on L. pneumophila infection. Expression of CaM in the wild-type strain abolished the activity of the SidE family, and ubiquitination of Rab33b in cells infected with this strain became undetectable (Fig. 6A, top, fourth lane). If SdjA can counteract the activity of SidJ, overexpression of this deglutamylase in this strain should restore Rab33b ubiquitination. Indeed, introducing a plasmid expressing SdjA into strain Lp02(pCaM) led to detectable Rab33b ubiquitination in infected cells (Fig. 6A, top, fifth lane). Expression of CaM and/or SdjA from multicopy plasmids did not interfere with the expression or translocation of endogenous SdeA and SidJ (Fig. 6A, middle and bottom), suggesting that Rab33b ubiquitination occurred in cells infected with strain Lp02(pCaM, pSdjA) is caused by increased activity of SdeA.

One function of SidEs during L. pneumophila infection is to recruit and modify the ER protein RTN4 by phosphoribosyl ubiquitination (32). We examined how SdjA impacts this phenotype by examining the association of RTN4 with the LCV formed by these L. pneumophila strains. Approximately 60% of the vacuoles harboring the wild-type strain stained positive for RTN4. Such recruitment was abolished by deleting members of the *sidE* family, which can be fully complemented by expressing SdeA from a plasmid (Fig. 6B). Akin to its inhibition of Rab33b ubiquitination (Fig. 1E), expression of CaM in the wild-type strain abolished its ability to recruit RTN4 (Fig. 6B), indicating inactivation of SidEs in bacterial cells by this cofactor. Importantly, in cells infected with strain Lp02(pCaM, pSdjA), recruitment of RTN4 by the bacterial phagosome significantly increased (Fig. 6B). These results suggest that SdjA restores the activity of SdeA, leading to the recruitment of RTN4 to the LCV.

Finally, whereas expression of CaM in the wild-type of L. pneumophila strain caused significant defects in intracellular replication, coexpression of SdjA in this strain markedly restored bacterial virulence, implying that SdjA alleviates the inhibitory effects imposed by SidJ-induced glutamylation of some members of the SidE family, particularly SdeA (Fig. 6C).

## DISCUSSION

The SidE effector family of L. pneumophila is required for optimal virulence in amoeba hosts (45, 48). These proteins catalyze phosphoribosyl ubiquitination of a large cohort of host proteins, including many that are essential for cell viability (26, 32, 37, 38). To prevent their potential excessive damage to host physiology, the bacterium has evolved two distinct mechanisms to regulate the activity of SidEs: first, the use of the phosphodiesterases DupA and DupB to specifically remove phosphoribosyl ubiquitin from modified proteins (37, 38), and second, the inhibition of the ADP-ribosyltransferase activity by the calmodulin-dependent glutamylase SidJ (39–42). Our results herein demonstrate that SdjA, the homolog of SidJ, is a bifunctional protein that distinctly regulates the activity of members of the SidE family.

SdjA satisfies the criteria for a CaM-dependent glutamylase against SdeB and SdeC, including effective suppression of their yeast toxicity, strong inhibition of their ubiquitin ligase activity in mammalian cells, the ability to inactivate their ADP-ribosyltransferase activity in ubiquitin activation, the ability to covalently attach glutamate moieties to the first glutamate residues of the ExE motif in their mART domain, and finally, the ability to inhibit substrate ubiquitination induced by SdeB and SdeC in cells infected with L. pneumophila (Fig. 1 and 4). To some extent, SdjA and SidJ are functionally redundant in their activities toward SdeB and SdeC.

The most unexpected activity of SdjA is its ability to antagonize the inhibitory effects of SidJ against SdeA. Several lines of evidence suggest that SdjA is also a deglutamylase that functions to remove glutamate from Glu-SdeA. First, SdjA effectively alleviated SidJ-mediated inhibition of SdeA activity in mammalian cells. Second, incubation of recombinant SdjA_251–807_ with inactive Glu-SdeA restored its ubiquitin ligase activity. Third, GST-tagged SdjA containing its degradation forms effectively removed radioactivity from [^14^C]Glu-SdeA produced by SidJ (Fig. 5). Finally, overexpression of SdjA appeared to restore the ubiquitin ligase activity of SdeA in a wild-type L. pneumophila strain expressing CaM (Fig. 6).

**FIG 7:**
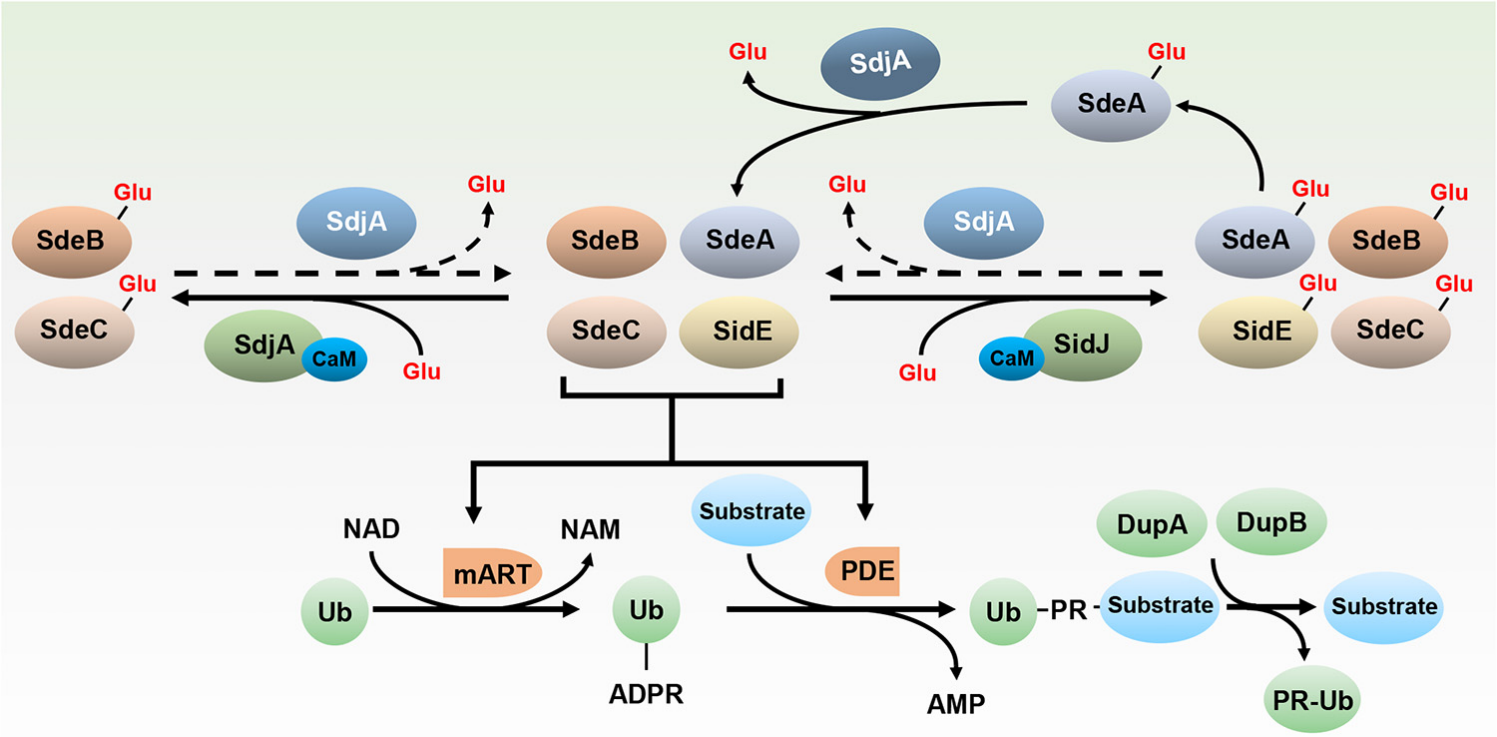
Summary diagram of the regulation of SidE activity. The NAD-dependent ubiquitin ligase activities of SidE, SdeA, SdeB, and SdeC are carried out by two sequential reactions catalyzed by mART and PDE activity, respectively. Modified substrates are returned to their original forms by DupA and DupB. The activity of each SidE family member is inhibited by SidJ, a glutamylase that is activated by CaM. In addition to inhibiting the ubiquitin ligase activity of SdeB and SdeC by a mechanism similar to that of SidJ, SdjA regulates the activity of SidJ by reversing the glutamylation on SdeA.

The deglutamylase activity of SdjA appears to be regulated by some yet-unrecognized mechanisms. Whereas full-length SdjA in complex with CaM expressed in E. coli did not have detectable deglutamylase activity against Glu-SdeA, such activity became robust in experiments in which SdjA tended to be degraded (Fig. 5; Fig. S4). It is likely that the deglutamylase activity seen in bacterial or mammalian cells in which SdjA was expressed either by its endogenous promoter or by transfection results from a fragment derived from spontaneous degradation. Apparently, based on the present results, the activity associated with SdjA expressed in cells is specific for SdeA. These results differ from those of SdjA_251–807_, a truncation mutant that indiscriminately removed glutamate from all members of the SidE family in biochemical reactions (Fig. 5B). A recent study revealed that the N-terminal domain (NTD) of SdjA is involved in recognizing members of the SidE family; replacement of the helix-turn-helix motif (HTH) in SdjA with that from SidJ made it able to modify SdeA by glutamylation (47). This HTH motif, and even the NTD, is absent in our truncation mutant SdjA_251–807_, which may explain its indiscriminate deglutamylation activity toward all members of the SidE family. However, it remains to be investigated how SdjA recognizes SidE family proteins in the absence of the NTD. For the *in cellulo* deglutamylase activity of SdjA, we speculate that SdjA may work with some yet-unidentified factors in eukaryotic cells to achieve its specificity toward SdeA. Another possibility is that the deglutamylase activity of SdjA does come from a degraded form of SdjA, yet the glutamylation activity of full-length SdjA toward SdeB and SdeC is so strong that it overwhelms the deglutamylation activity of the degraded form of SdjA in cells. The dual activity of SdjA adds further complexity to the regulation of the SidEs. In addition to the reversal of modified substrates by DupA and DupB (37, 38), the ubiquitin ligase activity is inhibited by SidJ (39–42) and for some members by both SidJ and SdjA, with the latter being able to alleviate the inhibitory effects of SidJ on SdeA (Fig. 7).

The dual activity of SdjA is reminiscent of MavC, the transglutaminase that under normal conditions induces protein cross-linking between ubiquitin and the E2 enzyme UBE2N (27). However, this enzyme also hydrolyzed its own product into UBE2N and Ub when high concentrations of protein were used (49, 50). MavC and MvcA, the enzyme that reverses the modification, are structurally similar with an identical catalytic center (34, 35, 49, 50). It has been proposed that the same catalytic mechanism is used for both the forward and reverse reactions and that it is the affinity between the product and the enzyme that dictates the outcome of the reaction (50). The removal of the glutamate moiety from Glu-SdeA requires the cleavage of an isopeptide bond. However, our attempts to identify residues in SdjA_251–807_ critical for its deglutamylase activity have not been successful. Notably, SdjA_251–807_ has completely lost the glutamylation activity against all members of the SidE family (Fig. S6), which is consistent with the notion that the NTD of SidJ/SdjA is essential for its glutamylation activity. Together with the observation that a SdjA mutant defective in the glutamylase activity is still active in deglutamylation (Fig. 5D), these results suggest that the two activities are separate and distinct, at least under conditions used for our biochemical reactions. Moreover, while CaM is essential for the glutamylation activity of SdjA, it is dispensable for the deglutamylation activity of SdjA. Future structure-based analysis will be instrumental in determining the exact catalytic mechanism for the peptidase activity of SdjA. Such comparative analysis may reveal how such subtle variations in structures are translated into drastic differences in functionality, which will extend our understanding of not only the relationship between structure and function but also protein evolution.

10.1128/mBio.02316-21.6 FIG S6 SdjA lacking the NTD does not exhibit glutamylation activity. The experiment was performed as for Fig. 4A except that SdjA was replaced by SdjA_251–807_. The glutamylation activity was evaluated by the loss of the ability to produce Ub-Rab33b, which did not occur in any of these reactions. Download FIG S6, PDF file, 0.9 MB. Copyright © 2021 Song et al. 2021 Song et al. https://creativecommons.org/licenses/by/4.0/ This content is distributed under the terms of the Creative Commons Attribution 4.0 International license.

## MATERIALS AND METHODS

### Bacterial strains, plasmids, and cell culture.

E. coli strains DH5α and XL1-Blue were used for molecular cloning, and strain BL21(DE3) was used for recombinant protein production. E. coli was grown on LB agar plates or in LB medium at 37°C. To maintain plasmids in E. coli, antibiotics were used at the following concentrations: ampicillin, 100 μg/ml; kanamycin, 30 μg/ml. All L. pneumophila strains were derived from the Philadelphia 1 strain Lp02 and the *dotA* mutant strain Lp03 and are listed in Table S1 (51). L. pneumophila was cultured in liquid *N*-(2-acetamido)-2-aminoethanesulfonic acid-buffered yeast extract medium (AYE) or on solid charcoal-buffered yeast extract medium (CYE). When necessary, thymidine was added to a final concentration of 0.2 g/ml. Plasmids derived from pZL507 (52) were maintained in L. pneumophila by thymidine autotrophy. Gene deletion in L. pneumophila was carried out as described previously (43). Additional methods are available in the supplemental material.

10.1128/mBio.02316-21.7 TABLE S1 Bacterial strains, plasmids, and primers used in this study. Download Table S1, PDF file, 0.06 MB. Copyright © 2021 Song et al. 2021 Song et al. https://creativecommons.org/licenses/by/4.0/ This content is distributed under the terms of the Creative Commons Attribution 4.0 International license.

10.1128/mBio.02316-21.8 TEXT S1 Additional materials and methods. Download Text S1, PDF file, 0.07 MB. Copyright © 2021 Song et al. 2021 Song et al. https://creativecommons.org/licenses/by/4.0/ This content is distributed under the terms of the Creative Commons Attribution 4.0 International license.
