# Supplementary material for: The Legionella Effector SdjA Is a Bifunctional Enzyme That Distinctly Regulates Phosphoribosyl Ubiquitination
Source: mBio. 2021 Sep 7;12(5):e02316-21. doi: 10.1128/mBio.02316-21 (PMC8546864; doi:10.1128/mBio.02316-21)
Supplement: FIG S1 [file mbio.02316-21-sf001.pdf]

```
SdjA/1-807    1 MFSYLDKLLDGIFGYQKSD---------TSQLST-----------STPPTLTTVP--------------------  35
SidJ/1-873    1 MFGFIKKVLD-FFGVDQSEDNPSETAVETTDVSTKIKTTDTTQEESSVKTKTVVPTQPGGSVKPETIAPDQQKKH  74

SdjA/1-807   36 ------------------------LKQEYFVKIGESETQDLGLLPVVSKRHNQSVPLEEIPFEDTRLELIELYIA  86
SidJ/1-873   75 QIKTETTTSTTKQKGPKVTLMDGHVKQYYFARRGETSTHDTSLPPPVKVLSGRSIPLKEIPFEATRNELVQIYLT 149

SdjA/1-807   87 LLKQCVLDEKLKSIPAQYLISHYLFIKTLAANEGNKGRKDLYLNLSQKVADYLEKNESKIWSMAVECAKTSEYPI 161
SidJ/1-873  150 SIDKLIKSNKLNSIPSQQIASHYLFLRSLANSETDGIKKNQILSLAKPLGTYLASKEPHVWKMINELIEKSEYPI 224

SdjA/1-807  162 VDWIKKHHLHFNFIRAFILDYNKKSLTHNQRAFMQQFRDSAAFFFPDQVYLAWLTQSYEPGSILNPMYRESRSTH 236
SidJ/1-873  225 IHYLKNNRAHSNFMLALIHEYHKEPLTKNQSAFVQKFRDSSVFLFPNPIYTAWLAHSYDEDSSFNPMFRERLSTN 299

SdjA/1-807  237 YYHANITDNLLLRTRPKQVNFGPQHFFQKGKGPVKNTYRFNINDGKLMRIQGRTLLFSTNKGNEVIAVKVQKKGE 311
SidJ/1-873  300 FYHSTLTDNLLLRTEPKEVTLSSEHHYKKEKGPIDSSFRYQMSSDRLLRIQGRTLLFSTPQ-NDVVAVKVQKKGE 373

SdjA/1-807  312 PQSALSNEFQMADYLLKHQRRLNLQSQLPTPLSQYSINRTEILEKCSKSPDFEKFKNLISDAKSLEIYVYKATPS 386
SidJ/1-873  374 PKSTLEEEFEMADYLLKHQRRLDVHSKLPQPLGQYSVKKSEILEISRGSLDFERFKTLIDDSKDLEVYVYKAPQS 448

SdjA/1-807  387 YFTYLHDKQQSFSQLTSSVQKNVHDLFVLLREGIVFPYLADMFHTHIDESKRSDKGRYQTLVELLSALQSQMGRL 461
SidJ/1-873  449 YFTYLHDKNQDLEDLTASVKTNVHDLFVLLREGIVFPQLADIFHTHFGEDEREDKGRYQALVQLLNVLQFQLGRI 523

SdjA/1-807  462 DKWQKAVEFVNLRASGIADLGDNLPLTSFLTVSDWTKHYHAELLTGVYHPSFLFLDKSSGSVRSLFNSRRKIFGN 536
SidJ/1-873  524 DKWQKAVEYVNLRSSGLADLGDSLPITSLFTSSDFTKHYFSELLTGGYHP--TFFDKSSGTANSLFTGKRRLFGN 596

SdjA/1-807  537 YLYLNIIAEYLLVIQLVIGCYGDKVTRKMNSKSK-AKVWEHLAELMFSSCAEAVNLITGMPKSRALAFLKQRANV 610
SidJ/1-873  597 YLYLNTIAEYLLVIQLTLGSYGDKVTRDMMDKPKKEAVWRELANVMFTSCAEAIHIMTGIPQSRALTLLKQRANI 671

SdjA/1-807  611 RKHTQQTSFWMTPDYSNLDRLSVQSQQSTLYPGESDYEINSDLISGVGLSLDGINQDLGDYNQASPLRELEKLLY 685
SidJ/1-873  672 EKHFRQTQFWMTPDYSKLDEDTLQMEQYSIYSGEPEYEFTDKLVSGVGLSVDGVHQDLGGYNRESPLRELEKLLY 746

SdjA/1-807  686 ATVTLIEGTQQLDKQFFQQLDETEKMILSAAGVDK--CYQAVAKLLDLARPGCQMQRRLAFTYYEMIKRIYPCS- 757
SidJ/1-873  747 ATVTLIEGTMQLDKEFFKQLEQVEKILSGEIKTDANSCFEAVAQLLDLARPGCHFQKRLVLSYYEEAKLKYPSAP 821

SdjA/1-807  758 -NPADVRFELVAKEEAIIKIQRFWREHKKENQSLEKGFDFDRNTSSSQSPL--                       807
SidJ/1-873  822 TDAYDSRFQVVARTNAAITIQRFWREARK-NLSEKSDIDSEKPESERTTDKRL                       873
```
