# Supplementary material for: The Legionella Effector SdjA Is a Bifunctional Enzyme That Distinctly Regulates Phosphoribosyl Ubiquitination
Source: mBio. 2021 Sep 7;12(5):e02316-21. doi: 10.1128/mBio.02316-21 (PMC8546864; doi:10.1128/mBio.02316-21)
Supplement: FIG S2 [file mbio.02316-21-sf002.pdf]

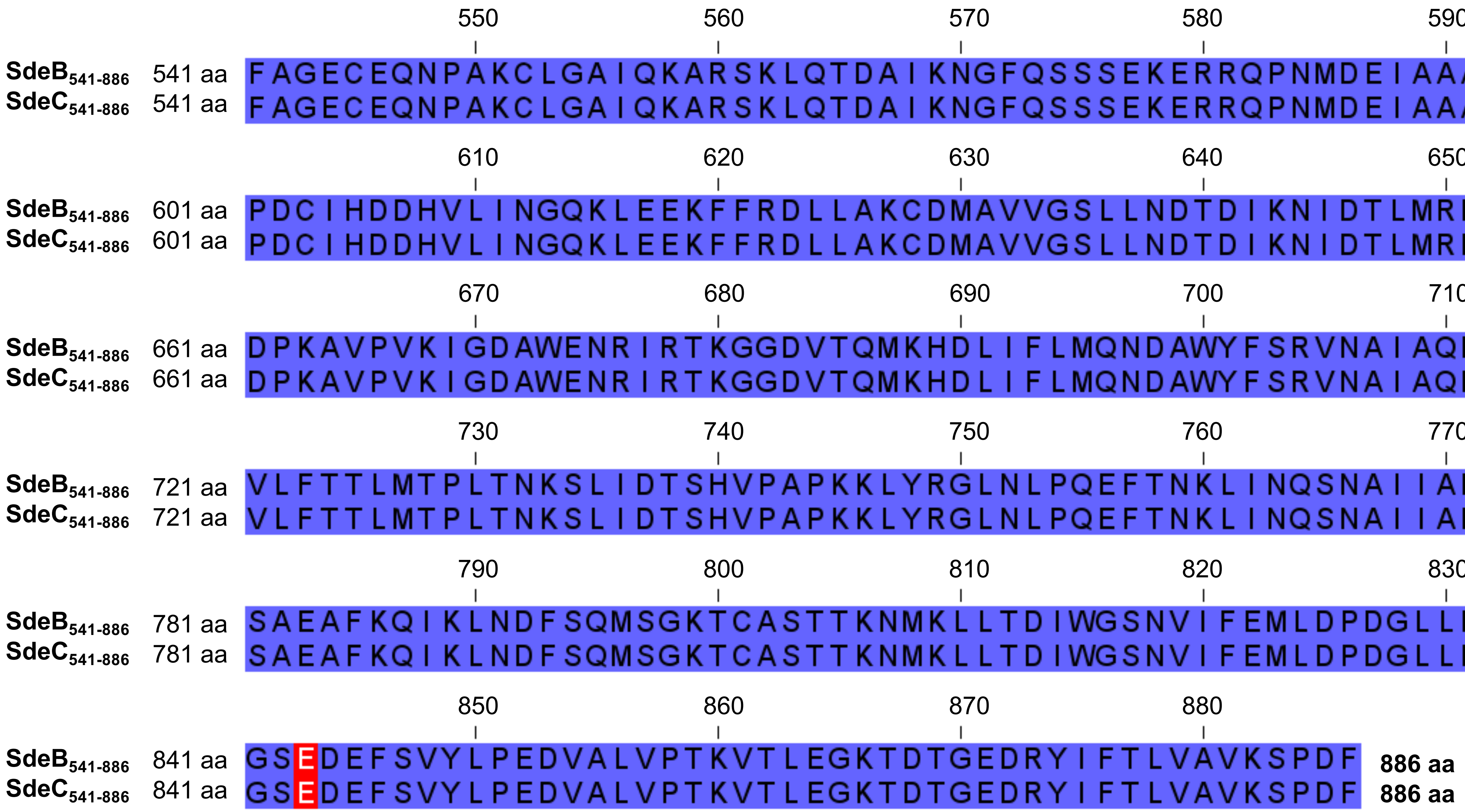
550 560 570 580 590
SdeB$_{541-886}$ 541 aa FAGECEQNPAKCLGAIQKARSKLQTDAIKNGFQSSSEKERRQPNMDEIAAARIIQQIMAN 600 aa
SdeC$_{541-886}$ 541 aa FAGECEQNPAKCLGAIQKARSKLQTDAIKNGFQSSSEKERRQPNMDEIAAARIIQQIMAN 600 aa
610 620 630 640 650
SdeB$_{541-886}$ 601 aa PDCIHDDHVLINGQKLEEKFFRDLLAKCDMAVVGSLLNDTDIKNIDTLMRHEKNTEFHST 660 aa
SdeC$_{541-886}$ 601 aa PDCIHDDHVLINGQKLEEKFFRDLLAKCDMAVVGSLLNDTDIKNIDTLMRHEKNTEFHST 660 aa
670 680 690 700 710
SdeB$_{541-886}$ 661 aa DPKAVPVKIGDAWENRIRTKGGDVTQMKHDLIFLMQNDAWYFSRVNAIAQNRDKGSNFKE 720 aa
SdeC$_{541-886}$ 661 aa DPKAVPVKIGDAWENRIRTKGGDVTQMKHDLIFLMQNDAWYFSRVNAIAQNRDKGSNFKE 720 aa
730 740 750 760 770
SdeB$_{541-886}$ 721 aa VLFTTLMTPLTNKSLIDTSHVPAPKKLYRGLNLPQEFTNKLINQSNAIIANTENTLFTDL 780 aa
SdeC$_{541-886}$ 721 aa VLFTTLMTPLTNKSLIDTSHVPAPKKLYRGLNLPQEFTNKLINQSNAIIANTENTLFTDL 780 aa
790 800 810 820 830
SdeB$_{541-886}$ 781 aa SAEAFKQIKLNDFSQMSGKTCASTTKNMKLLTDIWGSNVIFEMLDPDGLLHPKQVGTHMT 840 aa
SdeC$_{541-886}$ 781 aa SAEAFKQIKLNDFSQMSGKTCASTTKNMKLLTDIWGSNVIFEMLDPDGLLHPKQVGTHMA 840 aa
850 860 870 880
SdeB$_{541-886}$ 841 aa GSEDEFSVYLPEDVALVPTKVTLEGKTDTGEDRYIFTLVAVKSPDF 886 aa
SdeC$_{541-886}$ 841 aa GSEDEFSVYLPEDVALVPTKVTLEGKTDTGEDRYIFTLVAVKSPDF 886 aa
