# Supplementary figures and images for: The Legionella Effector SdjA Is a Bifunctional Enzyme That Distinctly Regulates Phosphoribosyl Ubiquitination
Source: mBio. 2021 Sep 7;12(5):e02316-21. doi: 10.1128/mBio.02316-21 (PMC8546864; doi:10.1128/mBio.02316-21)

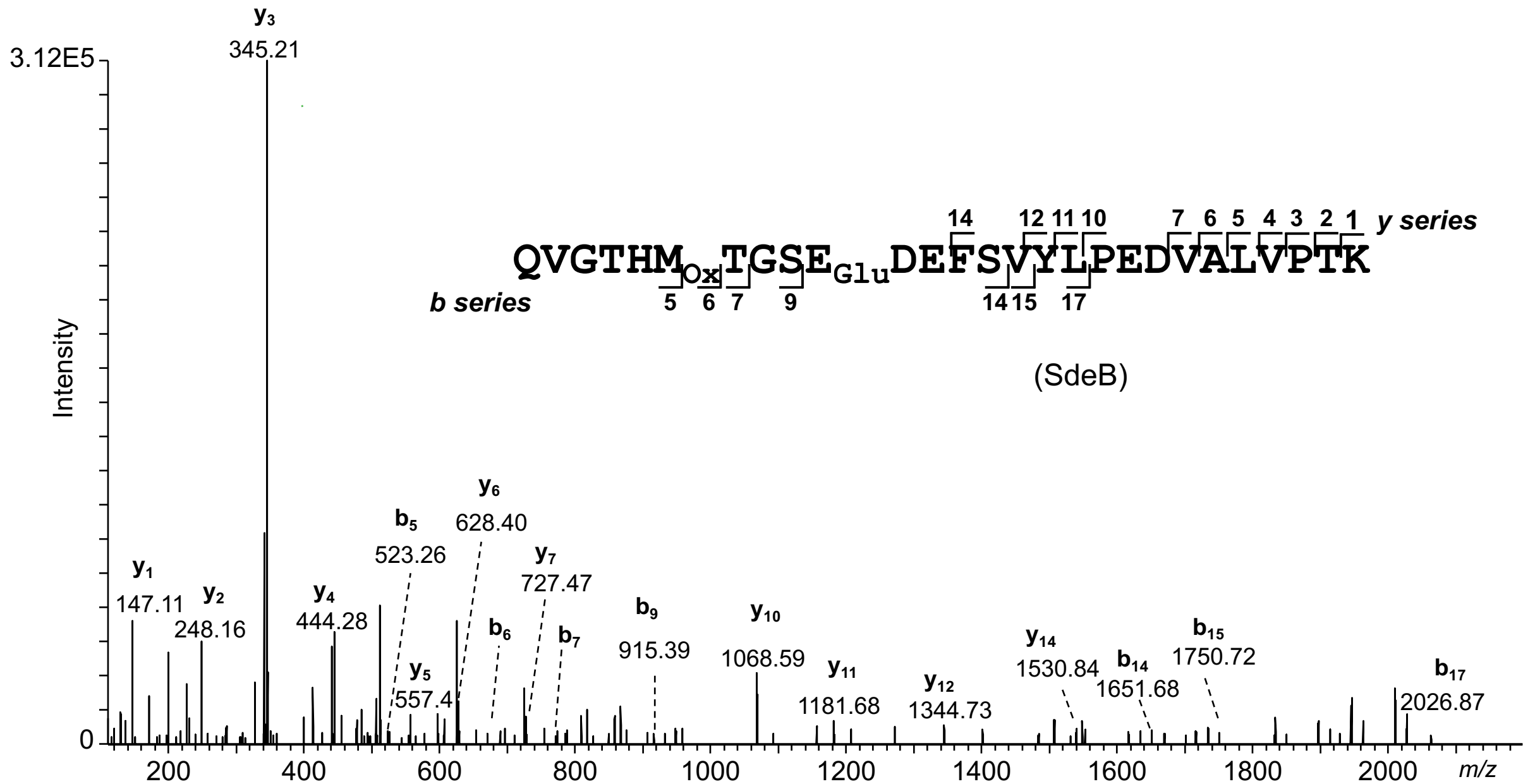

Supplement: FIG S3 [file mbio.02316-21-sf003.pdf]

|                         |   |   |   |   |   |   |   |   |
|-------------------------|---|---|---|---|---|---|---|---|
| SdeA                    | + | - | - | - | - | - | - | - |
| SdeA-Glu                | - | + | + | + | - | - | - | - |
| SdjA <sub>FL</sub> +CaM | - | - | + | - | + | - | + | - |
| Degraded SdjA           | - | - | - | + | - | + | - | + |
| Ub/NAD/Rab33b           | + | + | + | + | + | + | - | - |

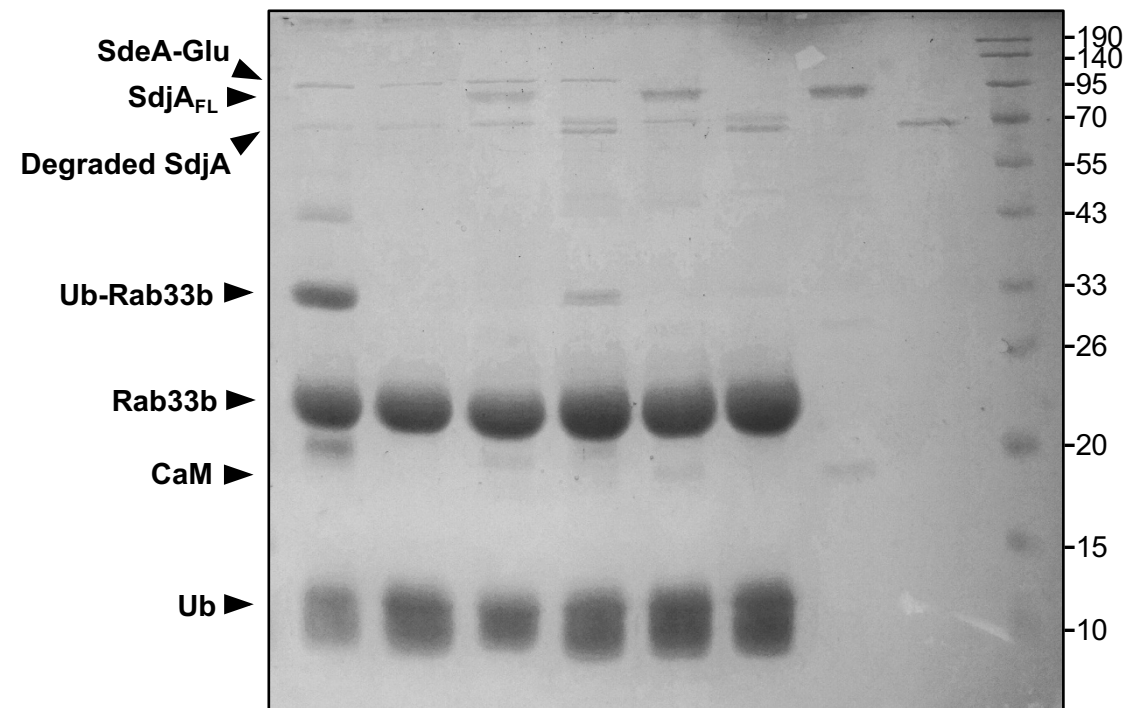

Supplement: FIG S4 [file mbio.02316-21-sf004.pdf]

**A**

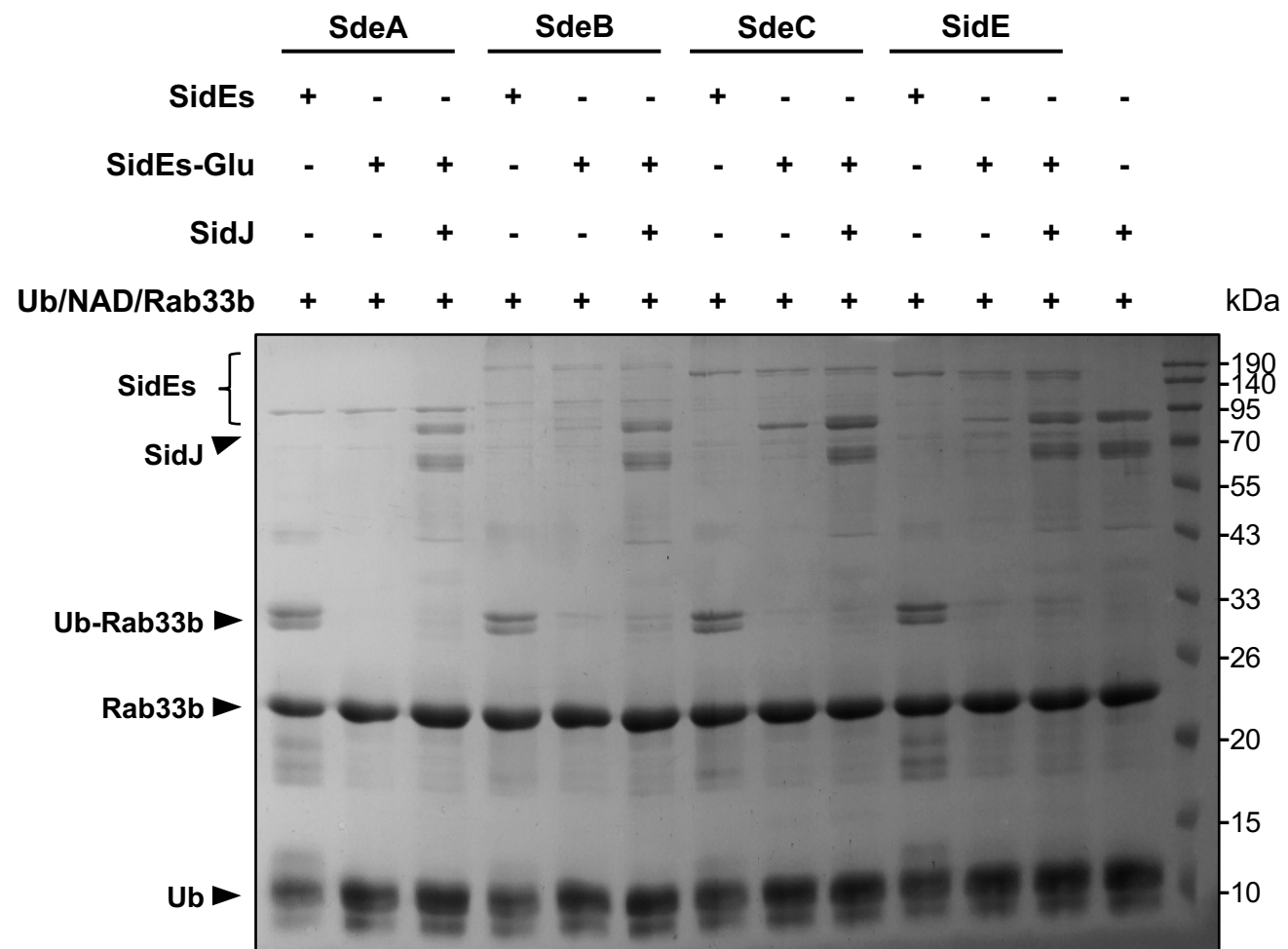

**B**

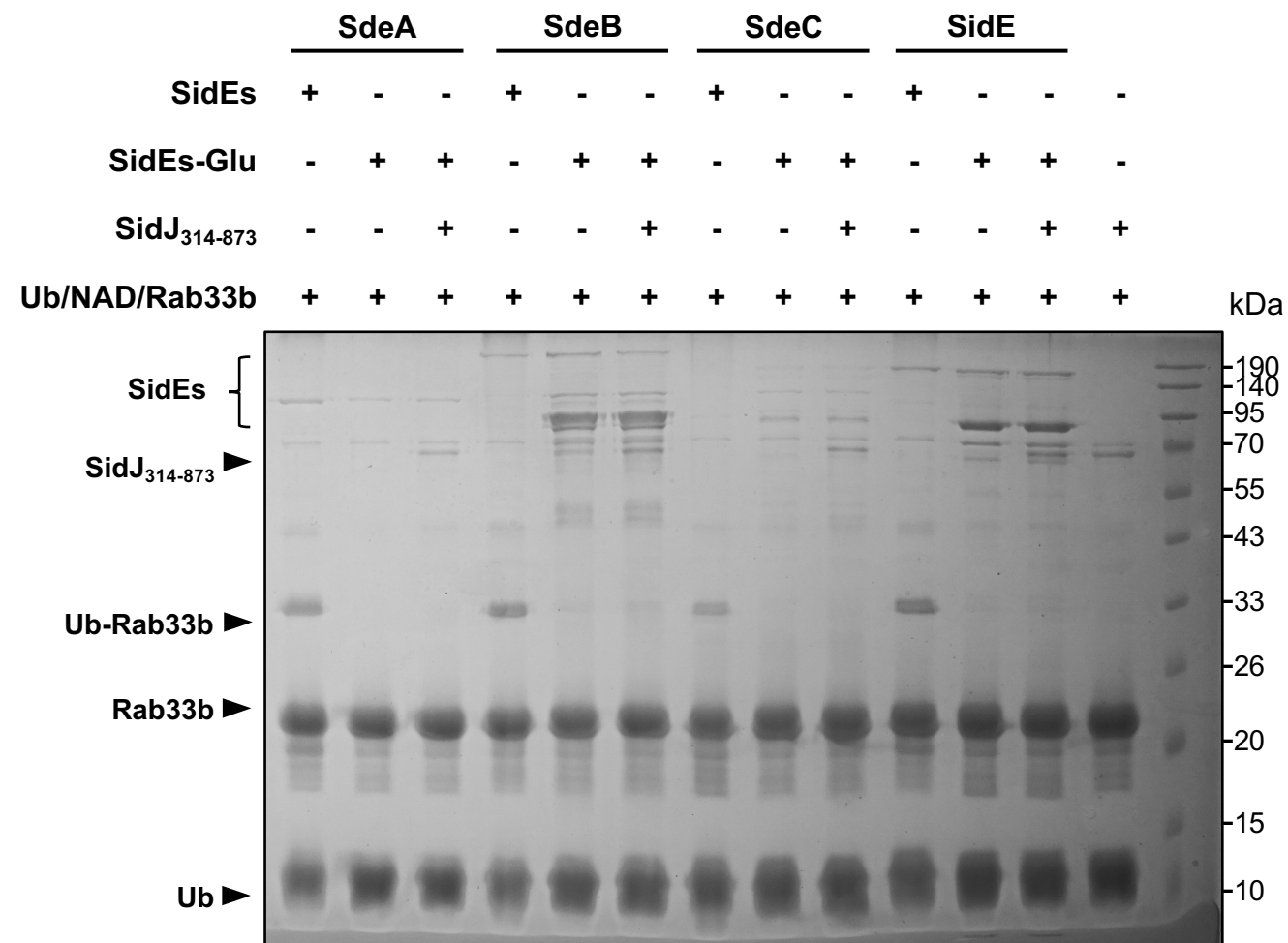

Supplement: FIG S5 [file mbio.02316-21-sf005.pdf]

Reaction2                      Ub   NAD   Rab33b   37 °C for 10 min

---

Reaction1                      37 °C for 2 h   (25 µL)

---

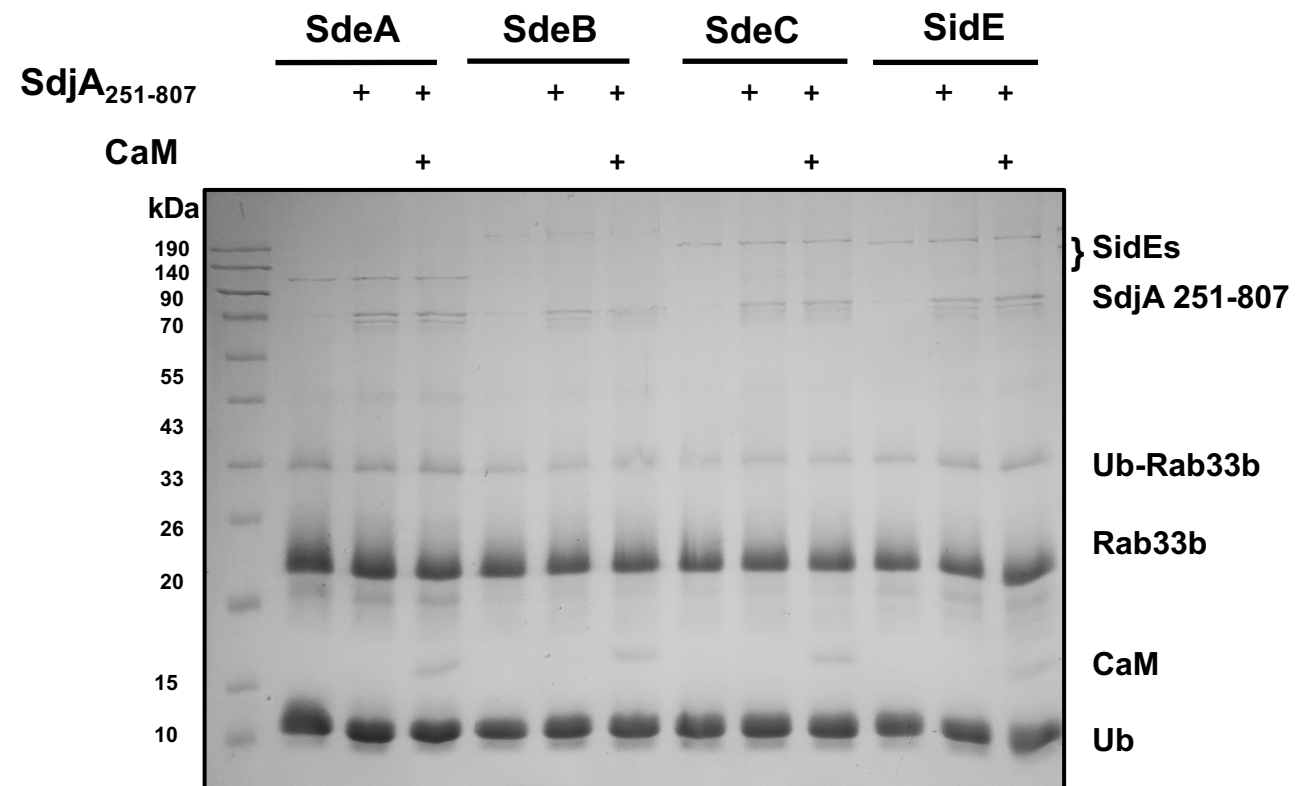

Supplement: FIG S6 [file mbio.02316-21-sf006.pdf]
