# Supplementary material for: The Legionella Effector SdjA Is a Bifunctional Enzyme That Distinctly Regulates Phosphoribosyl Ubiquitination
Source: mBio. 2021 Sep 7;12(5):e02316-21. doi: 10.1128/mBio.02316-21 (PMC8546864; doi:10.1128/mBio.02316-21)
Supplement: TABLE S1 [file mbio.02316-21-st001.pdf]

Table S1 Bacterial strains, plasmids and primers used in this study

Bacterial Strains	Source	Identifier
L. pneumophila (Philadelphia-1) LP02	(1)	N/A
L. pneumophila LP03	(1)	N/A
LP02Δ*sidEs*	(2)	N/A
LP02Δ*sidEs*(pZL507-SdeA)	(2)	N/A
LP02(pZL507-CaM)	This study	N/A
LP02(pZL507-CaM, pZLQ-SdjA)	This study	N/A
LP02Δ*sidJ*	(3)	N/A
LP02Δ*sidJ*Δ*sdjA*	(3)	N/A
LP02Δ*sidJ*(pZL507-CaM)	This study	N/A
LP02Δ*sidEs*Δ*sidJ*	This study	N/A
LP02Δ*sidEs*Δ*sidJ*(pZL507, pZLQ)	This study	N/A
LP02Δ*sidEs*Δ*sidJ*(pZL507-SdeA, pZLQ)	This study	N/A
LP02Δ*sidEs*Δ*sidJ*(pZL507-SdeA, pZLQ-CaM)	This study	N/A
LP02Δ*sidEs*Δ*sidJ*(pZL507-SdeC, pZLQ)	This study	N/A
LP02Δ*sidEs*Δ*sidJ*(pZL507-SdeC, pZLQ-CaM)	This study	N/A
E.coli BL21(DE3)	Transgen	CAT# CD601
E.coli XL1-Blue	Transgen	CAT# CD401

Plasmids	Source	Identifier	Tag	Experiments
pZL507	(4)	N/A	His	Fig. 1C/F
pZL507::*sdeA*	(2)	N/A	His	Fig. 1F, Fig. 6B
pZL507::*CaM*	This study	N/A	His	Fig. 1C, Fig. 6A/B
pZL507::*sdeC*	This study	N/A	His	Fig. 1F
pZLQ	(5)	N/A	Flag	Fig. 1F

pZLQ::*CaM*	This study	N/A	Flag	Fig. 1F
pZLQ::*sdjA*	This study	N/A	Flag	Fig. 6A/B
pAPH	This study	N/A	HA	N/A
pAPH::*sdeA*	This study	N/A	HA	Fig. 3B
pAPH::$sdeB_{1\text{-}1757}$	This study	N/A	HA	Fig. 3B
pAPH::*sdeC*	This study	N/A	HA	Fig. 3B
pAPH::$sidE_{222\text{-}1057}$	This study	N/A	HA	Fig. 3B
Paph::$sdeA_{mART}$	This study	N/A	HA	Fig. 3D
Paph::$sdeB_{mART}$	This study	N/A	HA	Fig. 3E
Paph::$sidE_{mART}$	This study	N/A	HA	Fig. 3D
pcDNA3.1:: 3×HA-*Ubaa*	(6)	N/A	3×HA	Fig. 3A, Fig. 5A
peGFPC1	Clontech	N/A	eGFP	Fig. 3B/D/E, Fig. 5A
peGFPC1::*sdeA*	(2)	N/A	eGFP	Fig. 3A, Fig. 5A
peGFPC1::$sdeB_{1\text{-}1757}$	This study	N/A	eGFP	Fig. 3A
peGFPC1::*sdeC*	This study	N/A	eGFP	Fig. 3A
peGFPC1::$sidE_{222\text{-}1057}$	This study	N/A	eGFP	Fig. 3A
peGFPC1::*sidJ*	This study	N/A	eGFP	Fig. 3D/E
peGFPC1::*sdjA*	This study	N/A	eGFP	Fig. 3B/D/E, Fig. 5A
pmCherry-C1	Clontech	CAT#632524	mCherry	Fig. 3A
pmCherry-C1::*sdjA*	This study	N/A	mCherry	Fig. 3A
pYES2/CT	Invitrogen	CAT#V825120	N/A	Fig. 2
pYES/CT::3×Flag-*sdeA*	This study	N/A	3×Flag	Fig. 2
pYES/CT::3×Flag-$sdeB_{1\text{-}1757}$	This study	N/A	3×Flag	Fig. 2
pYES/CT::3×Flag-*sdeC*	This study	N/A	3×Flag	Fig. 2
pYES/CT::3×Flag-$sidE_{222\text{-}1057}$	This study	N/A	3×Flag	Fig. 2
pCMV::4×Flag-Rab33B	(2)	N/A	4×Flag	Fig. 3B
pCMV::4×Flag-SidJ	(7)	N/A	4×Flag	Fig. 5A
p425GPD	(8)	N/A	N/A	Fig. 2
P425GPD::HA-*sdjA*	This study	N/A	HA	Fig. 2
pGEX6p-1	Cytiva	CAT# 28-9546-48	GST	N/A
pGEX6p-1::*sdeA*	This study	N/A	GST	Fig. 4B, Fig. 5C

pGEX6p-1::*sdeB*	This study	N/A	GST	Fig. 4B
pGEX6p-1::*sdeC*	This study	N/A	GST	Fig. 4B
pGEX6p-1::*sidE*	This study	N/A	GST	Fig. 4B
pGEX6p-1::*sdeA*$_{231-1190}$	This study	N/A	GST	Fig. 4A, Fig. 5B, Fig. S4, Fig. S5
pGEX6p-1::*sdeC*$_{1-1534}$	This study	N/A	GST	Fig. 4A, Fig. 5B, Fig. S5
pGEX6p-1::*sdjA*$_{37-782}$	This study	N/A	GST	Fig. 4A
pGEX6p-1::*hcaM*$_{1-149}$	This study	N/A	GST	Fig. S4
pET22b	Novagen	CAT#69337-3	His	N/A
pET22b/CT::*sidJ*$_{1-873}$	This study	N/A	His	Fig. S5A
pET22b/CT::*sidJ*$_{314-873}$	This study	N/A	His	Fig. S5B
pET28a	Novagen	CAT#69864	His	N/A
pET28a/CT::*sdeB*$_{1-1926}$	This study	N/A	His	Fig. 4A, Fig. 5B, Fig. S5
pET28a/CT::*sidE*$_{1-1496}$	This study	N/A	His	Fig. 4A, Fig. 5B, Fig. S5
pET28a/CT::*sdjA*$_{1-807}$	This study	N/A	His	Fig. 4B, Fig. 5C, Fig. S4
pET28a/CT::*sdjA*$_{251-807}$	This study	N/A	His	Fig. 5B
pET28a/CT::*hcaM*$_{1-149}$	This study	N/A	His	Fig. 4A
pET28a/NT::*hrab33b*$_{15-202}$	This study	N/A	His	Fig. 4A, Fig. 5B, Fig. S4, Fig. S5
pET28a/NT::*Ub*$_{1-76}$	This study	N/A	His	Fig. 3E, Fig. 4A, Fig. 5B, Fig. S4, Fig. S5
pQE30	Qiagen	CAT#32915	His	N/A
pQE30::*hrab33b*	(2)	N/A	His	Fig. 3E
pQE30::*sdeA*$_{E860A}$	(2)	N/A	His	Fig. 3E

**Primers**	**Sequence (Restriction enzyme sites are underlined)**	**Note**
pSL1001	cgcggatccatgtttagttatctggataaattat	*sdjA 5F BamHI*
pSL1002	acgcgtcgactcataaaggcgactgcga	*sdjA 3R SalI*
pSL1003	catgccatgggctttagttatctggataaa	*sdjA*$_{(1-807)}$ *5F NcoI*
pSL1004	ccgctcgagtaaaggcgactgcgatgaactagtgtt	*sdjA*$_{(1-807)}$ *3R XhoI*
pSL1005	tccaggggcccctgggatccatgaaacaagaatattttgta	*sdjA*$_{(37-782)}$ *5F BamHI*
pSL1006	tcacgatgcggccgctcgagttactctcgccaaaaacgctg	*sdjA*$_{(37-782)}$ *3R XhoI*
pSL1007	catgccatgggccgccccaaacaggtcaa	*sdjA*$_{(251-807)}$ *5F NcoI*

pSL1008	ccgctcgagtaaaggcgactgcgatgaactagtgtt	*sdjA*$_{(251-807)}$ *3R XhoI*
pSL1009	cgcggatccatgtttggtttcataaagaaagt	*sidJ* 5F BamHI
pSL1010	acgcgtcgactcacaaacgtttatcagtagt	*sidJ* 3R SalI
pSL1011	atacatatggaacctaaggaagtaac	*sidJ*$_{(314-873)}$ *5F NdeI*
pSL1012	acgcgtcgaccaaacgtttatcagtag	*sidJ*$_{(314-873)}$ *3R SalI*
pSL1013	cgcggatccatcagtttgggagaagcc	*sdeA 5F BamHI*
pSL1014	acgcgtcgacttaaaatcctatagttttttattggat	*sdeA 3R SalI*
pSL1015	ggaattccatatgggtttctctctgtacac	*sdeA*$_{(231-1190)}$ *5F NdeI*
pSL1016	ccgctcgagtcacgcgctaatttgtttgg	*sdeA*$_{(231-1190)}$ *3R XhoI*
pSL1017	cgcggatccaacagagcacgatgc	*sdeA*$_{mART}$ *5F BamHI*
pSL1018	acgcgtcgacttaaaagtcagggctttttacg	*sdeA*$_{mART}$ *3R SalI*
pSL1019	ggaagatctttgggagaaagtaaaatgcct	*sdeB*$_{(1-1757)}$ *5F BglII*
pSL1020	acgcgtcgacttatttactcttggattgcaaatcca	*sdeB*$_{(1-1757)}$ *3R SalI*
pSL1021	tttaagaaggagatataccatgggagaaagtaaaatgcctaaatatgta	*sdeB*$_{(1-1926)}$ *5F*
pSL1022	gtggtgctcgagtgcggccgcaaagtaaccactttctcggtgataggtatc	*sdeB*$_{(1-1926)}$ *3R*
pSL1023	cgcggatccatgcctaaatacgtagaagg	*sdeC 5F BamHI*
pSL1024	acgcgtcgacttagaaaccataccttatgtcat	*sdeC 3R SalI*
pSL1025	gggcccctgggatcccatatgcctaaatacgtaga	*sdeC*$_{(1-1534)}$ *5F NdeI*
pSL1026	ccgctcgagtagaccataccttatatcatcactttcttc	*sdeC*$_{(1-1534)}$ *3R XhoI*
pSL1027	cgcggatcctttgcaggagaatgtgaac	*sdeC*$_{mART}$ *5F BamHI*
pSL1028	acgcgtcgacttaaaagtcagggcttttaacag	*sdeC*$_{mART}$ *3R SalI*
pSL1029	cgcggatccatgttaactggaacatcacctaaag	*sidE*$_{(222-1057)}$ *5F BamHI*
pSL1030	ccggaattcttaattttggcattgagccaag	*sidE*$_{(222-1057)}$ *3R EcoRI*
pSL1031	ataagaatgcggccgcttaattttggcattgagccaag	*sidE*$_{(222-1057)}$ *3R NotI*
pSL1032	tttaagaaggagatataccatgcctaagtacgttgaagggatagaatta	*sidE*$_{(1-1496)}$ *5F*
pSL1033	gtggtgctcgagtgcggccgccaatcttaagagagttccttgtttttcctc	*sidE*$_{(1-1496)}$ *3R*
pSL1034	cgcggatccaacagagcacggtgt	*sidE*$_{mART}$ *5F BamHI*
pSL1035	ataagaatgcggccgcttagaaatcggggctttttacc	*sidE*$_{mART}$ *3R NotI*
pSL1036	catgccatggctgaccaactgactgaagagcaga	*hcaM*$_{(1-149)}$ *5F NcoI*
pSL1037	ccgctcgagctttgctgtcatcatttgtacaaactc	*hcaM*$_{(1-149)}$ *3R XhoI*
pSL1038	cgcggatccatggctgatcagct	*hcaM 5F BamHI*

pSL1039	acgcgtcgacttattttgcagtcatcatctgt	*hcaM 3R SalI*
pSL1040	ccgtcatatgcagatcttcgtgaa	*$Ub_{(1\text{-}76)}$ 5F NdeI*
pSL1041	ccgctcgagtcacccacctcggagac	*$Ub_{(1\text{-}76)}$ 3R XhoI*

N/A, not applicable.

1. Berger KH, Isberg RR. 1993. Two distinct defects in intracellular growth complemented by a single genetic locus in Legionella pneumophila. Mol Microbiol 7:7-19.
2. Qiu J, Sheedlo MJ, Yu K, Tan Y, Nakayasu ES, Das C, Liu X, Luo ZQ. 2016. Ubiquitination independent of E1 and E2 enzymes by bacterial effectors. Nature 533:120-4.
3. Liu Y, Luo ZQ. 2007. The Legionella pneumophila effector SidJ is required for efficient recruitment of endoplasmic reticulum proteins to the bacterial phagosome. Infect Immun 75:592-603.
4. Xu L, Shen X, Bryan A, Banga S, Swanson MS, Luo ZQ. 2010. Inhibition of host vacuolar H+-ATPase activity by a Legionella pneumophila effector. PLoS Pathog 6:e1000822.
5. Luo ZQ, Farrand SK. 1999. Signal-dependent DNA binding and functional domains of the quorum-sensing activator TraR as identified by repressor activity. Proc Natl Acad Sci U S A 96:9009-14.
6. Gan N, Nakayasu ES, Hollenbeck PJ, Luo ZQ. 2019. Legionella pneumophila inhibits immune signalling via MavC-mediated transglutaminase-induced ubiquitination of UBE2N. Nat Microbiol 4:134-143.
7. Gan N, Zhen X, Liu Y, Xu X, He C, Qiu J, Liu Y, Fujimoto GM, Nakayasu ES, Zhou B, Zhao L, Puvar K, Das C, Ouyang S, Luo ZQ. 2019. Regulation of phosphoribosyl ubiquitination by a calmodulin-dependent glutamylase. Nature 572:387-391.
8. Mumberg D, Muller R, Funk M. 1995. Yeast vectors for the controlled expression of heterologous proteins in different genetic backgrounds. Gene 156:119-22.
