# Supplementary material for: The Legionella Effector SdjA Is a Bifunctional Enzyme That Distinctly Regulates Phosphoribosyl Ubiquitination
Source: mBio. 2021 Sep 7;12(5):e02316-21. doi: 10.1128/mBio.02316-21 (PMC8546864; doi:10.1128/mBio.02316-21)
Supplement: TEXT S1 [file mbio.02316-21-s0001.pdf]

Supplemental methods

Restriction enzymes and T4 DNA ligase were purchased from New England Biolabs NEB. Gene was amplified by polymerase chain reaction (PCR) using Platinum™ SuperFi II Green PCR mix (Invitrogen, cat# 12369050). Site-directed mutagenesis was performed by the Quikchange kit (Agilent, cat# 600670) with primer pairs designed to introduce the desired mutations. The sequences of primers and plasmids used in this study are listed in **Table S1**. All substitution mutants were verified by double strand DNA sequencing.

HEK293T and HEK293 cells were purchased from ATCC and maintained in Dulbecco's modified minimal Eagle's medium (DMEM) supplemented with 10% Fetal Bovine Serum (FBS). Bone marrow-derived macrophages were isolated from 6- to 10-week-old female A/J mice (Jackson Lab) and were differentiated into macrophages using L-cell conditioned medium as described previously (1). Free of potential mycoplasma contamination in all mammalian cell lines were validated using PCR-based test (Sigma, cat# MP0025). pAPH-HA, a derivative of pVR1012 (2) suitable for expressing proteins with an amino HA tag and a carboxyl Flag tag.

Yeast toxicity assays

All yeast strains used in this study were derived from W303 (3); yeast was grown at 30°C in yeast extract, peptone, dextrose medium (YPD) medium or in appropriate amino acid dropout media in the presence of 2% of glucose or galactose as the sole carbon source. To test yeast toxicity, each member of the SidE family was cloned into pYES2CTA (Invitrogen) that harbors a galactose-inducible promoter and the resulting constructs were transformed into yeast strain W303 using the lithium acetate method (4). SdjA was cloned into p425GPD and individually transformed derivatives of W303 (3) that harbor members of the SidE family expressed from the P_{Gal} promoter. To determine the suppressor activity of SdjA, yeast cells cultured in liquid selection medium containing glucose were serially diluted (five-fold) in sterile water, and cells were spotted onto selective medium containing glucose or galactose. Images were acquired after 3-day incubation at 30°C.

Protein expression and purification

The genes of SidE, SdeA, SdeB, SdeC, SdeA (231-1190 residues), SdeC (1-1534 residues) and SdjA (37-782 residues) were amplified by PCR and cloned into pGEX6p-1 to produce GST-tagged fusion proteins with a PreScission Protease cleavage site between GST and the target proteins. The GST-tagged proteins were expressed in *E. coli* strain BL21 (DE3) and induced by 0.2 mM isopropyl-β-D-thiogalactopyranoside (IPTG) when the cell density reached an OD_{600} of 0.8. After growth at 16°C for 12 h, the cells were harvested and re-suspended in a lysis buffer (1×PBS, 2 mM DTT and 1 mM PMSF) and lysed by a cell homogenizer (JN-mini, JNBIO, Guangzhou, China). The cell lysates were centrifugated at 20,000*g* for 45 min at 4°C to remove cell debris. The supernatant was applied onto a self-packaged GST-affinity columns (2 mL glutathione Sepharose 4B; GE Healthcare) and unbound proteins were removed with wash buffer (lysis buffer plus 200 mM NaCl). The fusion protein was then digested with PreScission protease at 18°C for 2 h. GST-tagged proteins were similarly purified with glutathione beads and were eluted with 10 mM glutathione. Eluted protein was concentrated and further purified using a Superdex-200 (GE Healthcare) column equilibrated with a buffer containing 10 mM Tris–HCl pH 8.0, 200 mM NaCl, and 5 mM DTT. The purity of proteins was analyzed by SDS–PAGE followed by Coomassie brilliant blue staining.

The genes of ubiquitin (1-76 residues), human CaM (1-149 residues), human Rab33b (15-202 residues), SdjA (1-807 residues) and SdjA (251-807 residues) were cloned into pET28a, and SidJ (1-873 residues), SdeB (1-1926 residues) and SidE (1-1496 residues) were cloned into pET22b to express His_6-tagged proteins. The proteins were purified by Ni^{2+}-columns and gel filtration chromatography as described above. The full-length SdjA-CaM complex was obtained through co-expression of His_6-tagged SdjA and GST-tagged CaM. The protein complex was purified successively through Ni^{2+}-column and GST-affinity column. After digestion with PreScission protease, the SdjA-CaM complex was further purified through gel filtration chromatography.

Biochemical assays for glutamylation and de-glutamylation reactions

The glutamylase activity of SdjA was measured as we reported previously (5). Briefly, GST tagged SdeA or the other SidE family proteins (2 μg), GST-tagged SdjA (1 μg) and CaM (1 μM) were incubated in a 25-μL reaction system containing 50 mM Tris-

HCl (pH 7.5), 1 mM DTT, 5mM $MgCl_2$, 1 mM ATP and 1 μCi ^{14}C-L-glutamate (Perkin Elmer NEC290E050UC) for 2 h at 37°C.

For deglutamylation assays, relevant proteins were modified by SidJ and CaM in reactions for 2 h at 37°C. After adding 10 μL Ni^{2+}-NTA beads (Qiagen), the binding was allowed to proceed at 4°C for 14 h to remove His_6-sumo-SidJ and His_6-CaM. Supernatant containing ^{14}C-Glu-labeled GST-SdeA was collected by brief centrifugation and 1.0 μg, or 2.0 μg GST-SdjA was added to each reaction to a final volume of 25 μL. An equal volume of buffer was added to identical reactions as controls. The reactions were allowed to proceed for 2 h at 37°C prior to being terminated by adding 5 μL Laemmli buffer; samples boiled for 5 min were resolved by SDS-PAGE and were stained with Coomassie brilliant blue. For autoradiography, gels were dried, and signals were detected with X-ray films with a BioMax TranScreen LE (Kodak) for 2 weeks at −80°C.

Biochemical assays for phosphoribosyl ubiquitination

The glutamylation and de-glutamylation activity of SdjA was also investigated through its impact on the phosphoribosyl ubiquitination activity of SidE family proteins. For glutamylation assays, the reactions were divided into two steps. In the first step, reactions were performed in a mix containing 50 mM Tris-HCl pH 7.5, 50 mM NaCl, 1 mM DTT, 5 mM $MgCl_2$, 1 mM ATP, and 1.6 mM L-Glu. A typical 25 μL reaction contained 0.1 μM SdeA/SdeB/SdeC/SidE, 0.2 μM SdjA and 5 μM CaM. Reactions were allowed to proceed at 37°C for 2 h. In the second step, 0.8 mM NAD, 10 μM Rab33b and 37 μM Ub were added and the final volume of the reaction mix came to 30 μL, which was kept in 37°C for 10 min.

For de-glutamylation assays, the reactions were also divided into two steps. In the first step, the reactions were performed in a reaction mix containing 50 mM Tris-HCl pH 7.5, 50 mM NaCl, 1 mM DTT. A typical 14 μL reaction contained 0.1 μM SdeA/SdeB/SdeC/SidE or their glutamylated form, 0.2 μM SdjA or SidJ without CaM. Reactions were allowed to proceed at 37 °C for 30 min. In the second step, 0.8 mM NAD, 10 μM Rab33b and 37 μM Ub were added and the final volume came to 20 μL, which were kept in 37°C for 10 min. For the preparation of glutamylated SdeA, the

system was the same as the above glutamylation reaction system using SidJ and CaM to modify SdeA. After the reaction, His_6-tagged SidJ and CaM were removed by Ni^{2+}-columns, and Glu-SdeA was obtained from the flow through. For the preparation of Glu-SdeB/C/SidE, an anion exchange column was used for the purification of glutamylated proteins.

Transfection and Immunoprecipitation

HEK293T cells seeded in petri dishes or in wells of 6-well plates grown to 80% confluence were transfected using Lipofectamine 3000 (Invitrogen, cat# L3000150) according to the manufacture's protocol. Twenty-four h after transfection, cells were lysed using TBS buffer (50 mM Tris-HCl, 150 mM NaCl, PH 7.5) with 0.5% Triton X-100 for 10 min on ice. Cell lysates were centrifugated at 12,000*g* at 4°C for 10 min and the supernatant was then transferred to new tubes. After washed twice with lysis buffer, beads coated with Flag- (Sigma, cat# F2426) or HA-specific antibody (Sigma, cat# E6779) were added to the cleared cell lysates and the mixture was incubated on a rotatory shaker at 4°C overnight. After washed five times with the lysis buffer, Flag- or HA-tagged proteins bound to the resin were eluted with Flag peptide (Sigma, cat# F4799) or HA peptide (Sigma, cat# I2149), respectively. The elution was prepared for biochemical reaction or for immunoblotting.

Mass spectrometry analysis

Excised bands of interest were in-gel digested with trypsin using the Schevchenko protocol (6). Peptides were loaded into a 4 cm reversed-phase trapping column (in-house packed, 150 µm i.d. 5 µm Jupiter C18 particle from Phenomenex) connected to Waters NanoAcquity liquid chromatography (LC) system and washed at 5 µL/min for 10 min. Chromatography was performed on a 70 cm reversed-phase column (in-house packed, 75 µm i.d. 3 µm Jupiter C18 particle from Phenomenex) at 0.3 µL/min over 2 h. Peptides were eluted with a gradient of acetonitrile (solvent B) in water (solvent A) both containing 0.1% formic acid. The column was equilibrated with 1% B, and the gradient ramped to 8%-12%-30%-45%-95% B at 2-20-75-97-100 min, respectively. Eluting peptides were analyzed online on a Q Exactive HF-X mass spectrometer (Thermo Fisher Scientific) with

the ion source set to 2.2 kV for electrospray, 250 °C for capillary and RF lens at 30%. The top 12 most intense parent ions were submitted to high-energy collision dissociation with a normalized collision energy of 30. Each parent ion was fragmented once before being dynamically excluded for 45 seconds.

Data were analyzed using MaxQuant (v.1.6.17.0) (7)by searching tandem mass spectra against the Legionella pneumophila Philadelphia 01 protein sequences downloaded from the Uniprot Knowledgebase on April 13, 2021. Only peptides with specific trypsin digestion in both termini were considered for the identification. Methionine oxidation and glutamate glutamylation were set with variable modifications, with up to 2 modification sites allowed for each peptide. The remaining parameters of the software were set as the default option. Identified glutamylated spectra were manually inspected for matching b and y tandem mass fragments using Xcalibur v2.2 (Thermo Fisher Scientific).

Antibodies and immunoblotting

Samples from cells or bacteria lysates were prepared by adding 5×SDS loading buffer and heated at 95°C for 10 min. After resolved by SDS-PAGE electrophoresis, proteins were transferred onto polyvinylidene fluoride (PVDF) membranes (Pall Life Sciences). After blocking with 5% nonfat milk, the membranes were incubated with the appropriate primary antibodies at the indicated dilutions: α-Flag (Sigma, Cat# F1804, 1: 3000), α-GFP (Proteintech, cat# 50430-2-AP, 1:5000), α-mCherry (Proteintech, cat# 6765-1-AP, 1:3000), α-His (Sigma, cat# H1029, 1: 3,000), α-HA (Sigma, cat# H3663, 1: 3,000), α-ICDH (1: 10,000) (8), α-SdeA (1: 20,000) (9), α-SidJ (1: 5,000) (10), α-PGK (Abcam, cat# ab113687, 1:2,500) and α-GAPDH (Bioworld, cat# AP0063, 1: 10,000). Antibodies specific for SdjA were generated by immunization of rabbits with purified His_6-SdjA using a standard procedure (Jiaxuan Biotechnology Co., LTD, Beijing, China) and were used at 1:1000. After washed 3 times, the membranes were incubated with appropriate IRDye- or HRP-labeled secondary antibodies and the signals were detected and analyzed by an Odyssey CLx system (LI-COR) or ChemiDoc MP system (Bio-Rad).

Bacterial infection, immunostaining, image and bioinformatic analysis

For infection experiments, *L. pneumophila* strains were grown in AYE broth to the post-exponential growth phase (OD_{600}=3.3-3.8). If necessary, isopropyl β-D-1-thiogalactopyranoside (IPTG) was added into the broth at a final concentration of 0.2 mM for another 4 h at 37°C before infection. To determine the intracellular growth in *D. discoideum*, cells were infected with relevant *L. pneumophila* at a multiplicity of infection (MOI) of 0.05. 2 h after adding the bacteria, the samples were washed using warm PBS to remove the extracellular bacteria and then maintained in 22°C. At the indicated time points, cells were lysed with 0.2% saponin and appropriately diluted amounts of the lysates were plated on CYE plates. The counts of bacteria colonies were calculated to evaluate the fold growth after 4-day incubation at 37°C. To determine the impact of the infection on Rab33B ubiquitination, HEK293 cells were transfected with plasmids harboring 4xFlag-Rab33B and FcγRII receptor (11) 24 h before infection with the indicated bacterial strains. 2 h after infection, Rab33B were immunoprecipitated from the infected cells followed by immunoblotting analysis.

Sequence analysis and alignment were performed with Jalview (12) downloaded from www.jalview.org

For RTN4 immunostaining, mouse (A/J) bone marrow-derived macrophages were infected with relevant *L. pneumophila* strains at an MOI of 10 for 2 h. The procedure of immunostaining was as described (13). Briefly, cells were fixed with 4% paraformaldehyde and permeabilized using 0.1% Triton X-100. After blocking in 4% goat serum, samples were stained with rat anti-*Legionella* antibody (1:10,000), Hoechst DNA dye (1:10,000; Invitrogen) and rabbit anti-Rtn4 antibody (1:250; Life Span Biosciences), followed by incubated with appropriate secondary antibodies conjugated to specific fluorescence dyes. The images were taken using an Olympus IX-83 fluorescence microscope. To quantitate RTN4 recruitment, the proportion of Rtn4-positive vacuoles per strain was calculated over at least 150 bacterial vacuoles from each sample. All quantitative image analyses were performed in a double-blind manner.

Data quantitation, statistical analyses

A semi-quantitative analysis of the immunoblotting results was performed using the Image J software (1.8.0_172) provided by the NIH. Student's *t*-test was used to compare the mean levels between two groups each with at least three independent samples.

References:

1. Conover GM, Derre I, Vogel JP, Isberg RR. 2003. The Legionella pneumophila LidA protein: a translocated substrate of the Dot/Icm system associated with maintenance of bacterial integrity. Mol Microbiol 48:305-21.
2. Wang SH, Wang A, Liu PP, Zhang WY, Du J, Xu S, Liu GC, Zheng BS, Huan C, Zhao K, Yu XF. 2018. Divergent Pathogenic Properties of Circulating Coxsackievirus A6 Associated with Emerging Hand, Foot, and Mouth Disease. J Virol 92.
3. Tan Y, Arnold RJ, Luo ZQ. 2011. Legionella pneumophila regulates the small GTPase Rab1 activity by reversible phosphorylcholination. Proc Natl Acad Sci U S A 108:21212-7.
4. Gietz RD, Schiestl RH, Willems AR, Woods RA. 1995. Studies on the transformation of intact yeast cells by the LiAc/SS-DNA/PEG procedure. Yeast 11:355-60.
5. Gan N, Zhen X, Liu Y, Xu X, He C, Qiu J, Liu Y, Fujimoto GM, Nakayasu ES, Zhou B, Zhao L, Puvar K, Das C, Ouyang S, Luo ZQ. 2019. Regulation of phosphoribosyl ubiquitination by a calmodulin-dependent glutamylase. Nature 572:387-391.
6. Shevchenko A, Tomas H, Havlis J, Olsen JV, Mann M. 2006. In-gel digestion for mass spectrometric characterization of proteins and proteomes. Nat Protoc 1:2856-60.
7. Tyanova S, Temu T, Cox J. 2016. The MaxQuant computational platform for mass spectrometry-based shotgun proteomics. Nat Protoc 11:2301-2319.
8. Xu L, Shen X, Bryan A, Banga S, Swanson MS, Luo ZQ. 2010. Inhibition of host vacuolar H+-ATPase activity by a Legionella pneumophila effector. PLoS Pathog 6:e1000822.
9. Qiu J, Sheedlo MJ, Yu K, Tan Y, Nakayasu ES, Das C, Liu X, Luo ZQ. 2016. Ubiquitination independent of E1 and E2 enzymes by bacterial effectors. Nature 533:120-124.
10. Liu Y, Luo ZQ. 2007. The Legionella pneumophila effector SidJ is required for efficient recruitment of endoplasmic reticulum proteins to the bacterial phagosome. Infect Immun 75:592-603.
11. Qiu J, Sheedlo MJ, Yu K, Tan Y, Nakayasu ES, Das C, Liu X, Luo ZQ. 2016. Ubiquitination independent of E1 and E2 enzymes by bacterial effectors. Nature 533:120-4.
12. Waterhouse AM, Procter JB, Martin DM, Clamp M, Barton GJ. 2009. Jalview Version 2--a multiple sequence alignment editor and analysis workbench. Bioinformatics 25:1189-91.
13. Haenssler E, Ramabhadran V, Murphy CS, Heidtman MI, Isberg RR. 2015. Endoplasmic Reticulum Tubule Protein Reticulon 4 Associates with the Legionella

pneumophila Vacuole and with Translocated Substrate Ceg9. Infect Immun 83:3479-89.
